# Measurement of exclusive ${{{\uprho _{}^{}} _{}^{}}{{\left( {770}\right) }{}_{}^{}}} ^{0}$ photoproduction in ultraperipheral pPb collisions at $\sqrt{\smash [b]{s_{_{\mathrm {NN}}}}} = 5.02\,\text {Te}\text {V}$

**DOI:** 10.1140/epjc/s10052-019-7202-9

**Published:** 2019-08-21

**Authors:** A. M. Sirunyan, A. Tumasyan, W. Adam, F. Ambrogi, E. Asilar, T. Bergauer, J. Brandstetter, M. Dragicevic, J. Erö, A. Escalante Del Valle, M. Flechl, R. Frühwirth, V. M. Ghete, J. Hrubec, M. Jeitler, N. Krammer, I. Krätschmer, D. Liko, T. Madlener, I. Mikulec, N. Rad, H. Rohringer, J. Schieck, R. Schöfbeck, M. Spanring, D. Spitzbart, A. Taurok, W. Waltenberger, J. Wittmann, C.-E. Wulz, M. Zarucki, V. Chekhovsky, V. Mossolov, J. Suarez Gonzalez, E. A. De Wolf, D. Di Croce, X. Janssen, J. Lauwers, M. Pieters, M. Van De Klundert, H. Van Haevermaet, P. Van Mechelen, N. Van Remortel, S. Abu Zeid, F. Blekman, J. D’Hondt, I. De Bruyn, J. De Clercq, K. Deroover, G. Flouris, D. Lontkovskyi, S. Lowette, I. Marchesini, S. Moortgat, L. Moreels, Q. Python, K. Skovpen, S. Tavernier, W. Van Doninck, P. Van Mulders, I. Van Parijs, D. Beghin, B. Bilin, H. Brun, B. Clerbaux, G. De Lentdecker, H. Delannoy, B. Dorney, G. Fasanella, L. Favart, R. Goldouzian, A. Grebenyuk, A. K. Kalsi, T. Lenzi, J. Luetic, L. Moureaux, N. Postiau, E. Starling, L. Thomas, C. Vander Velde, P. Vanlaer, D. Vannerom, Q. Wang, T. Cornelis, D. Dobur, A. Fagot, M. Gul, I. Khvastunov, D. Poyraz, C. Roskas, D. Trocino, M. Tytgat, W. Verbeke, B. Vermassen, M. Vit, N. Zaganidis, H. Bakhshiansohi, O. Bondu, S. Brochet, G. Bruno, C. Caputo, P. David, C. Delaere, M. Delcourt, B. Francois, A. Giammanco, G. Krintiras, V. Lemaitre, A. Magitteri, A. Mertens, M. Musich, K. Piotrzkowski, A. Saggio, M. Vidal Marono, S. Wertz, J. Zobec, F. L. Alves, G. A. Alves, L. Brito, G. Correia Silva, C. Hensel, A. Moraes, M. E. Pol, P. Rebello Teles, E. Belchior Batista Das Chagas, W. Carvalho, J. Chinellato, E. Coelho, E. M. Da Costa, G. G. Da Silveira, D. De Jesus Damiao, C. De Oliveira Martins, S. Fonseca De Souza, H. Malbouisson, D. Matos Figueiredo, M. Melo De Almeida, C. Mora Herrera, L. Mundim, H. Nogima, W. L. Prado Da Silva, L. J. Sanchez Rosas, A. Santoro, A. Sznajder, M. Thiel, E. J. Tonelli Manganote, F. Torres Da Silva De Araujo, A. Vilela Pereira, S. Ahuja, C. A. Bernardes, L. Calligaris, T. R. Fernandez Perez Tomei, E. M. Gregores, P. G. Mercadante, S. F. Novaes, Sandra S. Padula, D. Romero Abad, A. Aleksandrov, R. Hadjiiska, P. Iaydjiev, A. Marinov, M. Misheva, M. Rodozov, M. Shopova, G. Sultanov, A. Dimitrov, L. Litov, B. Pavlov, P. Petkov, W. Fang, X. Gao, L. Yuan, M. Ahmad, J. G. Bian, G. M. Chen, H. S. Chen, M. Chen, Y. Chen, C. H. Jiang, D. Leggat, H. Liao, Z. Liu, F. Romeo, S. M. Shaheen, A. Spiezia, J. Tao, C. Wang, Z. Wang, E. Yazgan, H. Zhang, J. Zhao, Y. Ban, G. Chen, A. Levin, J. Li, L. Li, Q. Li, Y. Mao, S. J. Qian, D. Wang, Z. Xu, Y. Wang, C. Avila, A. Cabrera, C. A. Carrillo Montoya, L. F. Chaparro Sierra, C. Florez, C. F. González Hernández, M. A. Segura Delgado, B. Courbon, N. Godinovic, D. Lelas, I. Puljak, T. Sculac, Z. Antunovic, M. Kovac, V. Brigljevic, D. Ferencek, K. Kadija, B. Mesic, A. Starodumov, T. Susa, M. W. Ather, A. Attikis, M. Kolosova, G. Mavromanolakis, J. Mousa, C. Nicolaou, F. Ptochos, P. A. Razis, H. Rykaczewski, M. Finger, M. Finger, E. Ayala, E. Carrera Jarrin, A. Ellithi Kamel, M. A. Mahmoud, E. Salama, S. Bhowmik, A. Carvalho Antunes De Oliveira, R. K. Dewanjee, K. Ehataht, M. Kadastik, M. Raidal, C. Veelken, P. Eerola, H. Kirschenmann, J. Pekkanen, M. Voutilainen, J. Havukainen, J. K. Heikkilä, T. Järvinen, V. Karimäki, R. Kinnunen, T. Lampén, K. Lassila-Perini, S. Laurila, S. Lehti, T. Lindén, P. Luukka, T. Mäenpää, H. Siikonen, E. Tuominen, J. Tuominiemi, T. Tuuva, M. Besancon, F. Couderc, M. Dejardin, D. Denegri, J. L. Faure, F. Ferri, S. Ganjour, A. Givernaud, P. Gras, G. Hamel de Monchenault, P. Jarry, C. Leloup, E. Locci, J. Malcles, G. Negro, J. Rander, A. Rosowsky, M. Ö. Sahin, M. Titov, A. Abdulsalam, C. Amendola, I. Antropov, F. Beaudette, P. Busson, C. Charlot, R. Granier de Cassagnac, I. Kucher, S. Lisniak, A. Lobanov, J. Martin Blanco, M. Nguyen, C. Ochando, G. Ortona, P. Paganini, P. Pigard, R. Salerno, J. B. Sauvan, Y. Sirois, A. G. Stahl Leiton, A. Zabi, A. Zghiche, J.-L. Agram, J. Andrea, D. Bloch, J.-M. Brom, E. C. Chabert, V. Cherepanov, C. Collard, E. Conte, J.-C. Fontaine, D. Gelé, U. Goerlach, M. Jansová, A.-C. Le Bihan, N. Tonon, P. Van Hove, S. Gadrat, S. Beauceron, C. Bernet, G. Boudoul, N. Chanon, R. Chierici, D. Contardo, P. Depasse, H. El Mamouni, J. Fay, L. Finco, S. Gascon, M. Gouzevitch, G. Grenier, B. Ille, F. Lagarde, I. B. Laktineh, H. Lattaud, M. Lethuillier, L. Mirabito, A. L. Pequegnot, S. Perries, A. Popov, V. Sordini, M. Vander Donckt, S. Viret, S. Zhang, A. Khvedelidze, I. Bagaturia, C. Autermann, L. Feld, M. K. Kiesel, K. Klein, M. Lipinski, M. Preuten, M. P. Rauch, C. Schomakers, J. Schulz, M. Teroerde, B. Wittmer, V. Zhukov, A. Albert, D. Duchardt, M. Endres, M. Erdmann, T. Esch, R. Fischer, S. Ghosh, A. Güth, T. Hebbeker, C. Heidemann, K. Hoepfner, H. Keller, S. Knutzen, L. Mastrolorenzo, M. Merschmeyer, A. Meyer, P. Millet, S. Mukherjee, T. Pook, M. Radziej, H. Reithler, M. Rieger, F. Scheuch, A. Schmidt, D. Teyssier, G. Flügge, O. Hlushchenko, B. Kargoll, T. Kress, A. Künsken, T. Müller, A. Nehrkorn, A. Nowack, C. Pistone, O. Pooth, H. Sert, A. Stahl, M. Aldaya Martin, T. Arndt, C. Asawatangtrakuldee, I. Babounikau, K. Beernaert, O. Behnke, U. Behrens, A. Bermúdez Martínez, D. Bertsche, A. A. Bin Anuar, K. Borras, V. Botta, A. Campbell, P. Connor, C. Contreras-Campana, F. Costanza, V. Danilov, A. De Wit, M. M. Defranchis, C. Diez Pardos, D. Domínguez Damiani, G. Eckerlin, T. Eichhorn, A. Elwood, E. Eren, E. Gallo, A. Geiser, J. M. Grados Luyando, A. Grohsjean, P. Gunnellini, M. Guthoff, M. Haranko, A. Harb, J. Hauk, H. Jung, M. Kasemann, J. Keaveney, C. Kleinwort, J. Knolle, D. Krücker, W. Lange, A. Lelek, T. Lenz, K. Lipka, W. Lohmann, R. Mankel, I.-A. Melzer-Pellmann, A. B. Meyer, M. Meyer, M. Missiroli, G. Mittag, J. Mnich, V. Myronenko, S. K. Pflitsch, D. Pitzl, A. Raspereza, M. Savitskyi, P. Saxena, P. Schütze, C. Schwanenberger, R. Shevchenko, A. Singh, N. Stefaniuk, H. Tholen, O. Turkot, A. Vagnerini, G. P. Van Onsem, R. Walsh, Y. Wen, K. Wichmann, C. Wissing, O. Zenaiev, R. Aggleton, S. Bein, L. Benato, A. Benecke, V. Blobel, M. Centis Vignali, T. Dreyer, E. Garutti, D. Gonzalez, J. Haller, A. Hinzmann, A. Karavdina, G. Kasieczka, R. Klanner, R. Kogler, N. Kovalchuk, S. Kurz, V. Kutzner, J. Lange, D. Marconi, J. Multhaup, M. Niedziela, D. Nowatschin, A. Perieanu, A. Reimers, O. Rieger, C. Scharf, P. Schleper, S. Schumann, J. Schwandt, J. Sonneveld, H. Stadie, G. Steinbrück, F. M. Stober, M. Stöver, D. Troendle, A. Vanhoefer, B. Vormwald, M. Akbiyik, C. Barth, M. Baselga, S. Baur, E. Butz, R. Caspart, T. Chwalek, F. Colombo, W. De Boer, A. Dierlamm, N. Faltermann, B. Freund, M. Giffels, M. A. Harrendorf, F. Hartmann, S. M. Heindl, U. Husemann, F. Kassel, I. Katkov, S. Kudella, H. Mildner, S. Mitra, M. U. Mozer, Th. Müller, M. Plagge, G. Quast, K. Rabbertz, M. Schröder, I. Shvetsov, G. Sieber, H. J. Simonis, R. Ulrich, S. Wayand, M. Weber, T. Weiler, S. Williamson, C. Wöhrmann, R. Wolf, G. Anagnostou, G. Daskalakis, T. Geralis, A. Kyriakis, D. Loukas, G. Paspalaki, I. Topsis-Giotis, G. Karathanasis, S. Kesisoglou, P. Kontaxakis, A. Panagiotou, N. Saoulidou, E. Tziaferi, K. Vellidis, K. Kousouris, I. Papakrivopoulos, G. Tsipolitis, I. Evangelou, C. Foudas, P. Gianneios, P. Katsoulis, P. Kokkas, S. Mallios, N. Manthos, I. Papadopoulos, E. Paradas, J. Strologas, F. A. Triantis, D. Tsitsonis, M. Bartók, M. Csanad, N. Filipovic, P. Major, M. I. Nagy, G. Pasztor, O. Surányi, G. I. Veres, G. Bencze, C. Hajdu, D. Horvath, Á. Hunyadi, F. Sikler, T. Á. Vámi, V. Veszpremi, G. Vesztergombi, N. Beni, S. Czellar, J. Karancsi, A. Makovec, J. Molnar, Z. Szillasi, P. Raics, Z. L. Trocsanyi, B. Ujvari, S. Choudhury, J. R. Komaragiri, P. C. Tiwari, S. Bahinipati, C. Kar, P. Mal, K. Mandal, A. Nayak, D. K. Sahoo, S. K. Swain, S. Bansal, S. B. Beri, V. Bhatnagar, S. Chauhan, R. Chawla, N. Dhingra, R. Gupta, A. Kaur, A. Kaur, M. Kaur, S. Kaur, R. Kumar, P. Kumari, M. Lohan, A. Mehta, K. Sandeep, S. Sharma, J. B. Singh, G. Walia, A. Bhardwaj, B. C. Choudhary, R. B. Garg, M. Gola, S. Keshri, Ashok Kumar, S. Malhotra, M. Naimuddin, P. Priyanka, K. Ranjan, Aashaq Shah, R. Sharma, R. Bhardwaj, M. Bharti, R. Bhattacharya, S. Bhattacharya, U. Bhawandeep, D. Bhowmik, S. Dey, S. Dutt, S. Dutta, S. Ghosh, K. Mondal, S. Nandan, A. Purohit, P. K. Rout, A. Roy, S. Roy Chowdhury, S. Sarkar, M. Sharan, B. Singh, S. Thakur, P. K. Behera, R. Chudasama, D. Dutta, V. Jha, V. Kumar, P. K. Netrakanti, L. M. Pant, P. Shukla, T. Aziz, M. A. Bhat, S. Dugad, G. B. Mohanty, N. Sur, B. Sutar, RavindraKumar Verma, S. Banerjee, S. Bhattacharya, S. Chatterjee, P. Das, M. Guchait, Sa. Jain, S. Karmakar, S. Kumar, M. Maity, G. Majumder, K. Mazumdar, N. Sahoo, T. Sarkar, S. Chauhan, S. Dube, V. Hegde, A. Kapoor, K. Kothekar, S. Pandey, A. Rane, S. Sharma, S. Chenarani, E. Eskandari Tadavani, S. M. Etesami, M. Khakzad, M. Mohammadi Najafabadi, M. Naseri, F. Rezaei Hosseinabadi, B. Safarzadeh, M. Zeinali, M. Felcini, M. Grunewald, M. Abbrescia, C. Calabria, A. Colaleo, D. Creanza, L. Cristella, N. De Filippis, M. De Palma, A. Di Florio, F. Errico, L. Fiore, A. Gelmi, G. Iaselli, M. Ince, S. Lezki, G. Maggi, M. Maggi, G. Miniello, S. My, S. Nuzzo, A. Pompili, G. Pugliese, R. Radogna, A. Ranieri, G. Selvaggi, A. Sharma, L. Silvestris, R. Venditti, P. Verwilligen, G. Zito, G. Abbiendi, C. Battilana, D. Bonacorsi, L. Borgonovi, S. Braibant-Giacomelli, R. Campanini, P. Capiluppi, A. Castro, F. R. Cavallo, S. S. Chhibra, C. Ciocca, G. Codispoti, M. Cuffiani, G. M. Dallavalle, F. Fabbri, A. Fanfani, P. Giacomelli, C. Grandi, L. Guiducci, F. Iemmi, S. Marcellini, G. Masetti, A. Montanari, F. L. Navarria, A. Perrotta, F. Primavera, A. M. Rossi, T. Rovelli, G. P. Siroli, N. Tosi, S. Albergo, A. Di Mattia, R. Potenza, A. Tricomi, C. Tuve, G. Barbagli, K. Chatterjee, V. Ciulli, C. Civinini, R. D’Alessandro, E. Focardi, G. Latino, P. Lenzi, M. Meschini, S. Paoletti, L. Russo, G. Sguazzoni, D. Strom, L. Viliani, L. Benussi, S. Bianco, F. Fabbri, D. Piccolo, F. Ferro, F. Ravera, E. Robutti, S. Tosi, A. Benaglia, A. Beschi, L. Brianza, F. Brivio, V. Ciriolo, S. Di Guida, M. E. Dinardo, S. Fiorendi, S. Gennai, A. Ghezzi, P. Govoni, M. Malberti, S. Malvezzi, A. Massironi, D. Menasce, L. Moroni, M. Paganoni, D. Pedrini, S. Ragazzi, T. Tabarelli de Fatis, S. Buontempo, N. Cavallo, A. Di Crescenzo, A. Di Crescenzo, F. Fabozzi, F. Fienga, G. Galati, A. O. M. Iorio, W. A. Khan, L. Lista, S. Meola, P. Paolucci, C. Sciacca, E. Voevodina, P. Azzi, N. Bacchetta, D. Bisello, A. Boletti, A. Bragagnolo, R. Carlin, P. Checchia, M. Dall’Osso, P. De Castro Manzano, T. Dorigo, U. Dosselli, F. Gasparini, U. Gasparini, A. Gozzelino, S. Lacaprara, P. Lujan, M. Margoni, A. T. Meneguzzo, J. Pazzini, P. Ronchese, R. Rossin, F. Simonetto, A. Tiko, E. Torassa, M. Zanetti, P. Zotto, G. Zumerle, A. Braghieri, A. Magnani, P. Montagna, S. P. Ratti, V. Re, M. Ressegotti, C. Riccardi, P. Salvini, I. Vai, P. Vitulo, L. Alunni Solestizi, M. Biasini, G. M. Bilei, C. Cecchi, D. Ciangottini, L. Fanò, P. Lariccia, R. Leonardi, E. Manoni, G. Mantovani, V. Mariani, M. Menichelli, A. Rossi, A. Santocchia, D. Spiga, K. Androsov, P. Azzurri, G. Bagliesi, L. Bianchini, T. Boccali, L. Borrello, R. Castaldi, M. A. Ciocci, R. Dell’Orso, G. Fedi, F. Fiori, L. Giannini, A. Giassi, M. T. Grippo, F. Ligabue, E. Manca, G. Mandorli, A. Messineo, F. Palla, A. Rizzi, P. Spagnolo, R. Tenchini, G. Tonelli, A. Venturi, P. G. Verdini, L. Barone, F. Cavallari, M. Cipriani, N. Daci, D. Del Re, E. Di Marco, M. Diemoz, S. Gelli, E. Longo, B. Marzocchi, P. Meridiani, G. Organtini, F. Pandolfi, R. Paramatti, F. Preiato, S. Rahatlou, C. Rovelli, F. Santanastasio, N. Amapane, R. Arcidiacono, S. Argiro, M. Arneodo, N. Bartosik, R. Bellan, C. Biino, N. Cartiglia, F. Cenna, S. Cometti, M. Costa, R. Covarelli, N. Demaria, B. Kiani, C. Mariotti, S. Maselli, E. Migliore, V. Monaco, E. Monteil, M. Monteno, M. M. Obertino, L. Pacher, N. Pastrone, M. Pelliccioni, G. L. Pinna Angioni, A. Romero, M. Ruspa, R. Sacchi, K. Shchelina, V. Sola, A. Solano, D. Soldi, A. Staiano, S. Belforte, V. Candelise, M. Casarsa, F. Cossutti, G. Della Ricca, F. Vazzoler, A. Zanetti, D. H. Kim, G. N. Kim, M. S. Kim, J. Lee, S. Lee, S. W. Lee, C. S. Moon, Y. D. Oh, S. Sekmen, D. C. Son, Y. C. Yang, H. Kim, D. H. Moon, G. Oh, J. Goh, T. J. Kim, S. Cho, S. Choi, Y. Go, D. Gyun, S. Ha, B. Hong, Y. Jo, K. Lee, K. S. Lee, S. Lee, J. Lim, S. K. Park, Y. Roh, H. S. Kim, J. Almond, J. Kim, J. S. Kim, H. Lee, K. Lee, K. Nam, S. B. Oh, B. C. Radburn-Smith, S. h. Seo, U. K. Yang, H. D. Yoo, G. B. Yu, D. Jeon, H. Kim, J. H. Kim, J. S. H. Lee, I. C. Park, Y. Choi, C. Hwang, J. Lee, I. Yu, V. Dudenas, A. Juodagalvis, J. Vaitkus, I. Ahmed, Z. A. Ibrahim, M. A. B. Md Ali, F. Mohamad Idris, W. A. T. Wan Abdullah, M. N. Yusli, Z. Zolkapli, A. Castaneda Hernandez, J. A. Murillo Quijada, H. Castilla-Valdez, E. De La Cruz-Burelo, M. C. Duran-Osuna, I. Heredia-De La Cruz, R. Lopez-Fernandez, J. Mejia Guisao, R. I. Rabadan-Trejo, G. Ramirez-Sanchez, R. Reyes-Almanza, A. Sanchez-Hernandez, S. Carrillo Moreno, C. Oropeza Barrera, F. Vazquez Valencia, J. Eysermans, I. Pedraza, H. A. Salazar Ibarguen, C. Uribe Estrada, A. Morelos Pineda, D. Krofcheck, S. Bheesette, P. H. Butler, A. Ahmad, M. Ahmad, M. I. Asghar, Q. Hassan, H. R. Hoorani, A. Saddique, M. A. Shah, M. Shoaib, M. Waqas, H. Bialkowska, M. Bluj, B. Boimska, T. Frueboes, M. Górski, M. Kazana, K. Nawrocki, M. Szleper, P. Traczyk, P. Zalewski, K. Bunkowski, A. Byszuk, K. Doroba, A. Kalinowski, M. Konecki, J. Krolikowski, M. Misiura, M. Olszewski, A. Pyskir, M. Walczak, P. Bargassa, C. Beirão Da Cruz E Silva, A. Di Francesco, P. Faccioli, B. Galinhas, M. Gallinaro, J. Hollar, N. Leonardo, L. Lloret Iglesias, M. V. Nemallapudi, J. Seixas, G. Strong, O. Toldaiev, D. Vadruccio, J. Varela, P. Bunin, A. Golunov, I. Golutvin, V. Karjavin, V. Korenkov, G. Kozlov, A. Lanev, A. Malakhov, V. Matveev, V. V. Mitsyn, P. Moisenz, V. Palichik, V. Perelygin, S. Shmatov, V. Smirnov, V. Trofimov, B. S. Yuldashev, A. Zarubin, V. Zhiltsov, V. Golovtsov, Y. Ivanov, V. Kim, E. Kuznetsova, P. Levchenko, V. Murzin, V. Oreshkin, I. Smirnov, D. Sosnov, V. Sulimov, L. Uvarov, S. Vavilov, A. Vorobyev, Yu. Andreev, A. Dermenev, S. Gninenko, N. Golubev, A. Karneyeu, M. Kirsanov, N. Krasnikov, A. Pashenkov, D. Tlisov, A. Toropin, V. Epshteyn, V. Gavrilov, N. Lychkovskaya, V. Popov, I. Pozdnyakov, G. Safronov, A. Spiridonov, A. Stepennov, V. Stolin, M. Toms, E. Vlasov, A. Zhokin, T. Aushev, M. Chadeeva, P. Parygin, D. Philippov, S. Polikarpov, E. Popova, V. Rusinov, V. Andreev, M. Azarkin, I. Dremin, M. Kirakosyan, S. V. Rusakov, A. Terkulov, A. Baskakov, A. Belyaev, E. Boos, A. Ershov, A. Gribushin, L. Khein, V. Klyukhin, O. Kodolova, I. Lokhtin, O. Lukina, I. Miagkov, S. Obraztsov, S. Petrushanko, V. Savrin, A. Snigirev, V. Blinov, T. Dimova, L. Kardapoltsev, D. Shtol, Y. Skovpen, I. Azhgirey, I. Bayshev, S. Bitioukov, D. Elumakhov, A. Godizov, V. Kachanov, A. Kalinin, D. Konstantinov, P. Mandrik, V. Petrov, R. Ryutin, S. Slabospitskii, A. Sobol, S. Troshin, N. Tyurin, A. Uzunian, A. Volkov, A. Babaev, S. Baidali, P. Adzic, P. Cirkovic, D. Devetak, M. Dordevic, J. Milosevic, J. Alcaraz Maestre, A. Álvarez Fernández, I. Bachiller, M. Barrio Luna, J. A. Brochero Cifuentes, M. Cerrada, N. Colino, B. De La Cruz, A. Delgado Peris, C. Fernandez Bedoya, J. P. Fernández Ramos, J. Flix, M. C. Fouz, O. Gonzalez Lopez, S. Goy Lopez, J. M. Hernandez, M. I. Josa, D. Moran, A. Pérez-Calero Yzquierdo, J. Puerta Pelayo, I. Redondo, L. Romero, M. S. Soares, A. Triossi, C. Albajar, J. F. de Trocóniz, J. Cuevas, C. Erice, J. Fernandez Menendez, S. Folgueras, I. Gonzalez Caballero, J. R. González Fernández, E. Palencia Cortezon, V. Rodríguez Bouza, S. Sanchez Cruz, P. Vischia, J. M. Vizan Garcia, I. J. Cabrillo, A. Calderon, B. Chazin Quero, J. Duarte Campderros, M. Fernandez, P. J. Fernández Manteca, A. García Alonso, J. Garcia-Ferrero, G. Gomez, A. Lopez Virto, J. Marco, C. Martinez Rivero, P. Martinez Ruiz del Arbol, F. Matorras, J. Piedra Gomez, C. Prieels, T. Rodrigo, A. Ruiz-Jimeno, L. Scodellaro, N. Trevisani, I. Vila, R. Vilar Cortabitarte, D. Abbaneo, B. Akgun, E. Auffray, P. Baillon, A. H. Ball, D. Barney, J. Bendavid, M. Bianco, A. Bocci, C. Botta, E. Brondolin, T. Camporesi, M. Cepeda, G. Cerminara, E. Chapon, Y. Chen, G. Cucciati, D. d’Enterria, A. Dabrowski, V. Daponte, A. David, A. De Roeck, N. Deelen, M. Dobson, T. du Pree, M. Dünser, N. Dupont, A. Elliott-Peisert, P. Everaerts, F. Fallavollita, D. Fasanella, G. Franzoni, J. Fulcher, W. Funk, D. Gigi, A. Gilbert, K. Gill, F. Glege, M. Guilbaud, D. Gulhan, J. Hegeman, V. Innocente, A. Jafari, P. Janot, O. Karacheban, J. Kieseler, A. Kornmayer, M. Krammer, C. Lange, P. Lecoq, C. Lourenço, L. Malgeri, M. Mannelli, F. Meijers, J. A. Merlin, S. Mersi, E. Meschi, P. Milenovic, F. Moortgat, M. Mulders, J. Ngadiuba, S. Orfanelli, L. Orsini, F. Pantaleo, L. Pape, E. Perez, M. Peruzzi, A. Petrilli, G. Petrucciani, A. Pfeiffer, M. Pierini, F. M. Pitters, D. Rabady, A. Racz, T. Reis, G. Rolandi, M. Rovere, H. Sakulin, C. Schäfer, C. Schwick, M. Seidel, M. Selvaggi, A. Sharma, P. Silva, P. Sphicas, A. Stakia, J. Steggemann, M. Tosi, D. Treille, A. Tsirou, V. Veckalns, W. D. Zeuner, L. Caminada, K. Deiters, W. Erdmann, R. Horisberger, Q. Ingram, H. C. Kaestli, D. Kotlinski, U. Langenegger, T. Rohe, S. A. Wiederkehr, M. Backhaus, L. Bäni, P. Berger, N. Chernyavskaya, G. Dissertori, M. Dittmar, M. Donegà, C. Dorfer, C. Grab, C. Heidegger, D. Hits, J. Hoss, T. Klijnsma, W. Lustermann, R. A. Manzoni, M. Marionneau, M. T. Meinhard, F. Micheli, P. Musella, F. Nessi-Tedaldi, J. Pata, F. Pauss, G. Perrin, L. Perrozzi, S. Pigazzini, M. Quittnat, D. Ruini, D. A. Sanz Becerra, M. Schönenberger, L. Shchutska, V. R. Tavolaro, K. Theofilatos, M. L. Vesterbacka Olsson, R. Wallny, D. H. Zhu, T. K. Aarrestad, C. Amsler, D. Brzhechko, M. F. Canelli, A. De Cosa, R. Del Burgo, S. Donato, C. Galloni, T. Hreus, B. Kilminster, I. Neutelings, D. Pinna, G. Rauco, P. Robmann, D. Salerno, K. Schweiger, C. Seitz, Y. Takahashi, A. Zucchetta, Y. H. Chang, K. y. Cheng, T. H. Doan, Sh. Jain, R. Khurana, C. M. Kuo, W. Lin, A. Pozdnyakov, S. S. Yu, P. Chang, Y. Chao, K. F. Chen, P. H. Chen, W.-S. Hou, Arun Kumar, Y. y. Li, Y. F. Liu, R.-S. Lu, E. Paganis, A. Psallidas, A. Steen, J. f. Tsai, B. Asavapibhop, N. Srimanobhas, N. Suwonjandee, A. Bat, F. Boran, S. Cerci, S. Damarseckin, Z. S. Demiroglu, F. Dolek, C. Dozen, E. Eskut, S. Girgis, G. Gokbulut, Y. Guler, E. Gurpinar, I. Hos, C. Isik, E. E. Kangal, O. Kara, U. Kiminsu, M. Oglakci, G. Onengut, K. Ozdemir, S. Ozturk, A. Polatoz, D. Sunar Cerci, U. G. Tok, H. Topakli, S. Turkcapar, I. S. Zorbakir, C. Zorbilmez, B. Isildak, G. Karapinar, M. Yalvac, M. Zeyrek, I. O. Atakisi, E. Gülmez, M. Kaya, O. Kaya, S. Ozkorucuklu, S. Tekten, E. A. Yetkin, M. N. Agaras, S. Atay, A. Cakir, K. Cankocak, Y. Komurcu, S. Sen, B. Grynyov, L. Levchuk, F. Ball, L. Beck, J. J. Brooke, D. Burns, E. Clement, D. Cussans, O. Davignon, H. Flacher, J. Goldstein, G. P. Heath, H. F. Heath, L. Kreczko, D. M. Newbold, S. Paramesvaran, B. Penning, T. Sakuma, D. Smith, V. J. Smith, J. Taylor, A. Titterton, K. W. Bell, A. Belyaev, C. Brew, R. M. Brown, D. Cieri, D. J. A. Cockerill, J. A. Coughlan, K. Harder, S. Harper, J. Linacre, E. Olaiya, D. Petyt, C. H. Shepherd-Themistocleous, A. Thea, I. R. Tomalin, T. Williams, W. J. Womersley, G. Auzinger, R. Bainbridge, P. Bloch, J. Borg, S. Breeze, O. Buchmuller, A. Bundock, S. Casasso, D. Colling, L. Corpe, P. Dauncey, G. Davies, M. Della Negra, R. Di Maria, Y. Haddad, G. Hall, G. Iles, T. James, M. Komm, C. Laner, L. Lyons, A.-M. Magnan, S. Malik, A. Martelli, J. Nash, A. Nikitenko, V. Palladino, M. Pesaresi, A. Richards, A. Rose, E. Scott, C. Seez, A. Shtipliyski, G. Singh, M. Stoye, T. Strebler, S. Summers, A. Tapper, K. Uchida, T. Virdee, N. Wardle, D. Winterbottom, J. Wright, S. C. Zenz, J. E. Cole, P. R. Hobson, A. Khan, P. Kyberd, C. K. Mackay, A. Morton, I. D. Reid, L. Teodorescu, S. Zahid, K. Call, J. Dittmann, K. Hatakeyama, H. Liu, C. Madrid, B. Mcmaster, N. Pastika, C. Smith, R. Bartek, A. Dominguez, A. Buccilli, S. I. Cooper, C. Henderson, P. Rumerio, C. West, D. Arcaro, T. Bose, D. Gastler, D. Rankin, C. Richardson, J. Rohlf, L. Sulak, D. Zou, G. Benelli, X. Coubez, D. Cutts, M. Hadley, J. Hakala, U. Heintz, J. M. Hogan, K. H. M. Kwok, E. Laird, G. Landsberg, J. Lee, Z. Mao, M. Narain, S. Piperov, S. Sagir, R. Syarif, E. Usai, D. Yu, R. Band, C. Brainerd, R. Breedon, D. Burns, M. Calderon De La Barca Sanchez, M. Chertok, J. Conway, R. Conway, P. T. Cox, R. Erbacher, C. Flores, G. Funk, W. Ko, O. Kukral, R. Lander, C. Mclean, M. Mulhearn, D. Pellett, J. Pilot, S. Shalhout, M. Shi, D. Stolp, D. Taylor, K. Tos, M. Tripathi, Z. Wang, F. Zhang, M. Bachtis, C. Bravo, R. Cousins, A. Dasgupta, A. Florent, J. Hauser, M. Ignatenko, N. Mccoll, S. Regnard, D. Saltzberg, C. Schnaible, V. Valuev, E. Bouvier, K. Burt, R. Clare, J. W. Gary, S. M. A. Ghiasi Shirazi, G. Hanson, G. Karapostoli, E. Kennedy, F. Lacroix, O. R. Long, M. Olmedo Negrete, M. I. Paneva, W. Si, L. Wang, H. Wei, S. Wimpenny, B. R. Yates, J. G. Branson, S. Cittolin, M. Derdzinski, R. Gerosa, D. Gilbert, B. Hashemi, A. Holzner, D. Klein, G. Kole, V. Krutelyov, J. Letts, M. Masciovecchio, D. Olivito, S. Padhi, M. Pieri, M. Sani, V. Sharma, S. Simon, M. Tadel, A. Vartak, S. Wasserbaech, J. Wood, F. Würthwein, A. Yagil, G. Zevi Della Porta, N. Amin, R. Bhandari, J. Bradmiller-Feld, C. Campagnari, M. Citron, A. Dishaw, V. Dutta, M. Franco Sevilla, L. Gouskos, R. Heller, J. Incandela, A. Ovcharova, H. Qu, J. Richman, D. Stuart, I. Suarez, S. Wang, J. Yoo, D. Anderson, A. Bornheim, J. M. Lawhorn, H. B. Newman, T. Q. Nguyen, M. Spiropulu, J. R. Vlimant, R. Wilkinson, S. Xie, Z. Zhang, R. Y. Zhu, M. B. Andrews, T. Ferguson, T. Mudholkar, M. Paulini, M. Sun, I. Vorobiev, M. Weinberg, J. P. Cumalat, W. T. Ford, F. Jensen, A. Johnson, M. Krohn, S. Leontsinis, E. MacDonald, T. Mulholland, K. Stenson, K. A. Ulmer, S. R. Wagner, J. Alexander, J. Chaves, Y. Cheng, J. Chu, A. Datta, K. Mcdermott, N. Mirman, J. R. Patterson, D. Quach, A. Rinkevicius, A. Ryd, L. Skinnari, L. Soffi, S. M. Tan, Z. Tao, J. Thom, J. Tucker, P. Wittich, M. Zientek, S. Abdullin, M. Albrow, M. Alyari, G. Apollinari, A. Apresyan, A. Apyan, S. Banerjee, L. A. T. Bauerdick, A. Beretvas, J. Berryhill, P. C. Bhat, G. Bolla, K. Burkett, J. N. Butler, A. Canepa, G. B. Cerati, H. W. K. Cheung, F. Chlebana, M. Cremonesi, J. Duarte, V. D. Elvira, J. Freeman, Z. Gecse, E. Gottschalk, L. Gray, D. Green, S. Grünendahl, O. Gutsche, J. Hanlon, R. M. Harris, S. Hasegawa, J. Hirschauer, Z. Hu, B. Jayatilaka, S. Jindariani, M. Johnson, U. Joshi, B. Klima, M. J. Kortelainen, B. Kreis, S. Lammel, D. Lincoln, R. Lipton, M. Liu, T. Liu, J. Lykken, K. Maeshima, J. M. Marraffino, D. Mason, P. McBride, P. Merkel, S. Mrenna, S. Nahn, V. O’Dell, K. Pedro, C. Pena, O. Prokofyev, G. Rakness, L. Ristori, A. Savoy-Navarro, B. Schneider, E. Sexton-Kennedy, A. Soha, W. J. Spalding, L. Spiegel, S. Stoynev, J. Strait, N. Strobbe, L. Taylor, S. Tkaczyk, N. V. Tran, L. Uplegger, E. W. Vaandering, C. Vernieri, M. Verzocchi, R. Vidal, M. Wang, H. A. Weber, A. Whitbeck, D. Acosta, P. Avery, P. Bortignon, D. Bourilkov, A. Brinkerhoff, L. Cadamuro, A. Carnes, M. Carver, D. Curry, R. D. Field, S. V. Gleyzer, B. M. Joshi, J. Konigsberg, A. Korytov, P. Ma, K. Matchev, H. Mei, G. Mitselmakher, K. Shi, D. Sperka, J. Wang, S. Wang, Y. R. Joshi, S. Linn, A. Ackert, T. Adams, A. Askew, S. Hagopian, V. Hagopian, K. F. Johnson, T. Kolberg, G. Martinez, T. Perry, H. Prosper, A. Saha, V. Sharma, R. Yohay, M. M. Baarmand, V. Bhopatkar, S. Colafranceschi, M. Hohlmann, D. Noonan, M. Rahmani, T. Roy, F. Yumiceva, M. R. Adams, L. Apanasevich, D. Berry, R. R. Betts, R. Cavanaugh, X. Chen, S. Dittmer, O. Evdokimov, C. E. Gerber, D. A. Hangal, D. J. Hofman, K. Jung, J. Kamin, C. Mills, I. D. Sandoval Gonzalez, M. B. Tonjes, N. Varelas, H. Wang, X. Wang, Z. Wu, J. Zhang, M. Alhusseini, B. Bilki, W. Clarida, K. Dilsiz, S. Durgut, R. P. Gandrajula, M. Haytmyradov, V. Khristenko, J.-P. Merlo, A. Mestvirishvili, A. Moeller, J. Nachtman, H. Ogul, Y. Onel, F. Ozok, A. Penzo, C. Snyder, E. Tiras, J. Wetzel, B. Blumenfeld, A. Cocoros, N. Eminizer, D. Fehling, L. Feng, A. V. Gritsan, W. T. Hung, P. Maksimovic, J. Roskes, U. Sarica, M. Swartz, M. Xiao, C. You, A. Al-bataineh, P. Baringer, A. Bean, S. Boren, J. Bowen, A. Bylinkin, J. Castle, S. Khalil, A. Kropivnitskaya, D. Majumder, W. Mcbrayer, M. Murray, C. Rogan, S. Sanders, E. Schmitz, J. D. Tapia Takaki, Q. Wang, S. Duric, A. Ivanov, K. Kaadze, D. Kim, Y. Maravin, D. R. Mendis, T. Mitchell, A. Modak, A. Mohammadi, L. K. Saini, N. Skhirtladze, F. Rebassoo, D. Wright, A. Baden, O. Baron, A. Belloni, S. C. Eno, Y. Feng, C. Ferraioli, N. J. Hadley, S. Jabeen, G. Y. Jeng, R. G. Kellogg, J. Kunkle, A. C. Mignerey, F. Ricci-Tam, Y. H. Shin, A. Skuja, S. C. Tonwar, K. Wong, D. Abercrombie, B. Allen, V. Azzolini, A. Baty, G. Bauer, R. Bi, S. Brandt, W. Busza, I. A. Cali, M. D’Alfonso, Z. Demiragli, G. Gomez Ceballos, M. Goncharov, P. Harris, D. Hsu, M. Hu, Y. Iiyama, G. M. Innocenti, M. Klute, D. Kovalskyi, Y.-J. Lee, P. D. Luckey, B. Maier, A. C. Marini, C. Mcginn, C. Mironov, S. Narayanan, X. Niu, C. Paus, C. Roland, G. Roland, G. S. F. Stephans, K. Sumorok, K. Tatar, D. Velicanu, J. Wang, T. W. Wang, B. Wyslouch, S. Zhaozhong, A. C. Benvenuti, R. M. Chatterjee, A. Evans, P. Hansen, S. Kalafut, Y. Kubota, Z. Lesko, J. Mans, S. Nourbakhsh, N. Ruckstuhl, R. Rusack, J. Turkewitz, M. A. Wadud, J. G. Acosta, S. Oliveros, E. Avdeeva, K. Bloom, D. R. Claes, C. Fangmeier, F. Golf, R. Gonzalez Suarez, R. Kamalieddin, I. Kravchenko, J. Monroy, J. E. Siado, G. R. Snow, B. Stieger, A. Godshalk, C. Harrington, I. Iashvili, A. Kharchilava, D. Nguyen, A. Parker, S. Rappoccio, B. Roozbahani, G. Alverson, E. Barberis, C. Freer, A. Hortiangtham, D. M. Morse, T. Orimoto, R. Teixeira De Lima, T. Wamorkar, B. Wang, A. Wisecarver, D. Wood, S. Bhattacharya, O. Charaf, K. A. Hahn, N. Mucia, N. Odell, M. H. Schmitt, K. Sung, M. Trovato, M. Velasco, R. Bucci, N. Dev, M. Hildreth, K. Hurtado Anampa, C. Jessop, D. J. Karmgard, N. Kellams, K. Lannon, W. Li, N. Loukas, N. Marinelli, F. Meng, C. Mueller, Y. Musienko, M. Planer, A. Reinsvold, R. Ruchti, P. Siddireddy, G. Smith, S. Taroni, M. Wayne, A. Wightman, M. Wolf, A. Woodard, J. Alimena, L. Antonelli, B. Bylsma, L. S. Durkin, S. Flowers, B. Francis, A. Hart, C. Hill, W. Ji, T. Y. Ling, W. Luo, B. L. Winer, H. W. Wulsin, S. Cooperstein, P. Elmer, J. Hardenbrook, P. Hebda, S. Higginbotham, A. Kalogeropoulos, D. Lange, M. T. Lucchini, J. Luo, D. Marlow, K. Mei, I. Ojalvo, J. Olsen, C. Palmer, P. Piroué, J. Salfeld-Nebgen, D. Stickland, C. Tully, S. Malik, S. Norberg, A. Barker, V. E. Barnes, S. Das, L. Gutay, M. Jones, A. W. Jung, A. Khatiwada, B. Mahakud, D. H. Miller, N. Neumeister, C. C. Peng, H. Qiu, J. F. Schulte, J. Sun, F. Wang, R. Xiao, W. Xie, T. Cheng, J. Dolen, N. Parashar, Z. Chen, K. M. Ecklund, S. Freed, F. J. M. Geurts, M. Kilpatrick, W. Li, B. Michlin, B. P. Padley, J. Roberts, J. Rorie, W. Shi, Z. Tu, J. Zabel, A. Zhang, A. Bodek, P. de Barbaro, R. Demina, Y. t. Duh, J. L. Dulemba, C. Fallon, T. Ferbel, M. Galanti, A. Garcia-Bellido, J. Han, O. Hindrichs, A. Khukhunaishvili, K. H. Lo, P. Tan, R. Taus, M. Verzetti, R. Ciesielski, A. Agapitos, J. P. Chou, Y. Gershtein, T. A. Gómez Espinosa, E. Halkiadakis, M. Heindl, E. Hughes, S. Kaplan, R. Kunnawalkam Elayavalli, S. Kyriacou, A. Lath, R. Montalvo, K. Nash, M. Osherson, H. Saka, S. Salur, S. Schnetzer, D. Sheffield, S. Somalwar, R. Stone, S. Thomas, P. Thomassen, M. Walker, A. G. Delannoy, J. Heideman, G. Riley, K. Rose, S. Spanier, K. Thapa, O. Bouhali, A. Celik, M. Dalchenko, M. De Mattia, A. Delgado, S. Dildick, R. Eusebi, J. Gilmore, T. Huang, T. Kamon, S. Luo, R. Mueller, Y. Pakhotin, R. Patel, A. Perloff, L. Perniè, D. Rathjens, A. Safonov, A. Tatarinov, N. Akchurin, J. Damgov, F. De Guio, P. R. Dudero, S. Kunori, K. Lamichhane, S. W. Lee, T. Mengke, S. Muthumuni, T. Peltola, S. Undleeb, I. Volobouev, Z. Wang, S. Greene, A. Gurrola, R. Janjam, W. Johns, C. Maguire, A. Melo, H. Ni, K. Padeken, J. D. Ruiz Alvarez, P. Sheldon, S. Tuo, J. Velkovska, M. Verweij, Q. Xu, M. W. Arenton, P. Barria, B. Cox, R. Hirosky, M. Joyce, A. Ledovskoy, H. Li, C. Neu, T. Sinthuprasith, Y. Wang, E. Wolfe, F. Xia, R. Harr, P. E. Karchin, N. Poudyal, J. Sturdy, P. Thapa, S. Zaleski, M. Brodski, J. Buchanan, C. Caillol, D. Carlsmith, S. Dasu, L. Dodd, B. Gomber, M. Grothe, M. Herndon, A. Hervé, U. Hussain, P. Klabbers, A. Lanaro, A. Levine, K. Long, R. Loveless, T. Ruggles, A. Savin, N. Smith, W. H. Smith, N. Woods, CMS Collaboration

**Affiliations:** 1 Yerevan Physics Institute, Yerevan, Armenia; 2 Institut für Hochenergiephysik, Wien, Austria; 3 Institute for Nuclear Problems, Minsk, Belarus; 4 Universiteit Antwerpen, Antwerp, Belgium; 5 Vrije Universiteit Brussel, Brussels, Belgium; 6 Université Libre de Bruxelles, Brussels, Belgium; 7 Ghent University, Ghent, Belgium; 8 Université Catholique de Louvain, Louvain-la-Neuve, Belgium; 9 Centro Brasileiro de Pesquisas Fisicas, Rio de Janeiro, Brazil; 10 Universidade do Estado do Rio de Janeiro, Rio de Janeiro, Brazil; 11 Universidade Estadual Paulista, Universidade Federal do ABC, São Paulo, Brazil; 12 Institute for Nuclear Research and Nuclear Energy, Bulgarian Academy of Sciences, Sofia, Bulgaria; 13 University of Sofia, Sofia, Bulgaria; 14 Beihang University, Beijing, China; 15 Institute of High Energy Physics, Beijing, China; 16 State Key Laboratory of Nuclear Physics and Technology, Peking University, Beijing, China; 17 Tsinghua University, Beijing, China; 18 Universidad de Los Andes, Bogotá, Colombia; 19 University of Split, Faculty of Electrical Engineering, Mechanical Engineering and Naval Architecture, Split, Croatia; 20 University of Split, Faculty of Science, Split, Croatia; 21 Institute Rudjer Boskovic, Zagreb, Croatia; 22 University of Cyprus, Nicosia, Cyprus; 23 Charles University, Prague, Czech Republic; 24 Escuela Politecnica Nacional, Quito, Ecuador; 25 Universidad San Francisco de Quito, Quito, Ecuador; 26 Academy of Scientific Research and Technology of the Arab Republic of Egypt, Egyptian Network of High Energy Physics, Cairo, Egypt; 27 National Institute of Chemical Physics and Biophysics, Tallinn, Estonia; 28 Department of Physics, University of Helsinki, Helsinki, Finland; 29 Helsinki Institute of Physics, Helsinki, Finland; 30 Lappeenranta University of Technology, Lappeenranta, Finland; 31 IRFU, CEA, Université Paris-Saclay, Gif-sur-Yvette, France; 32 Laboratoire Leprince-Ringuet, Ecole polytechnique, CNRS/IN2P3, Université Paris-Saclay, Palaiseau, France; 33 Université de Strasbourg, CNRS, IPHC UMR 7178, Strasbourg, France; 34 Centre de Calcul de l’Institut National de Physique Nucleaire et de Physique des Particules, CNRS/IN2P3, Villeurbanne, France; 35 Université de Lyon, Université Claude Bernard Lyon 1, CNRS-IN2P3, Institut de Physique Nucléaire de Lyon, Villeurbanne, France; 36 Georgian Technical University, Tbilisi, Georgia; 37 Tbilisi State University, Tbilisi, Georgia; 38 RWTH Aachen University, I. Physikalisches Institut, Aachen, Germany; 39 RWTH Aachen University, III. Physikalisches Institut A, Aachen, Germany; 40 RWTH Aachen University, III. Physikalisches Institut B, Aachen, Germany; 41 Deutsches Elektronen-Synchrotron, Hamburg, Germany; 42 University of Hamburg, Hamburg, Germany; 43 Karlsruher Institut fuer Technologie, Karlsruhe, Germany; 44 Institute of Nuclear and Particle Physics (INPP), NCSR Demokritos, Aghia Paraskevi, Greece; 45 National and Kapodistrian University of Athens, Athens, Greece; 46 National Technical University of Athens, Athens, Greece; 47 University of Ioánnina, Ioannina, Greece; 48 MTA-ELTE Lendület CMS Particle and Nuclear Physics Group, Eötvös Loránd University, Budapest, Hungary; 49 Wigner Research Centre for Physics, Budapest, Hungary; 50 Institute of Nuclear Research ATOMKI, Debrecen, Hungary; 51 Institute of Physics, University of Debrecen, Debrecen, Hungary; 52 Indian Institute of Science (IISc), Bangalore, India; 53 National Institute of Science Education and Research, HBNI, Bhubaneswar, India; 54 Panjab University, Chandigarh, India; 55 University of Delhi, Delhi, India; 56 Saha Institute of Nuclear Physics, HBNI, Kolkata, India; 57 Indian Institute of Technology Madras, Madras, India; 58 Bhabha Atomic Research Centre, Mumbai, India; 59 Tata Institute of Fundamental Research-A, Mumbai, India; 60 Tata Institute of Fundamental Research-B, Mumbai, India; 61 Indian Institute of Science Education and Research (IISER), Pune, India; 62 Institute for Research in Fundamental Sciences (IPM), Tehran, Iran; 63 University College Dublin, Dublin, Ireland; 64 INFN Sezione di Bari, Università di Bari, Politecnico di Bari, Bari, Italy; 65 INFN Sezione di Bologna, Università di Bologna, Bologna, Italy; 66 INFN Sezione di Catania, Università di Catania, Catania, Italy; 67 INFN Sezione di Firenze, Università di Firenze, Firenze, Italy; 68 INFN Laboratori Nazionali di Frascati, Frascati, Italy; 69 INFN Sezione di Genova, Università di Genova, Genoa, Italy; 70 INFN Sezione di Milano-Bicocca, Università di Milano-Bicocca, Milan, Italy; 71 INFN Sezione di Napoli, Università di Napoli ‘Federico II’ , Naples, Italy, Università della Basilicata, Potenza, Italy, Università G. Marconi, Rome, Italy; 72 INFN Sezione di Padova, Università di Padova, Padova, Italy, Università di Trento, Trento, Italy; 73 INFN Sezione di Pavia, Università di Pavia, Pavia, Italy; 74 INFN Sezione di Perugia, Università di Perugia, Perugia, Italy; 75 INFN Sezione di Pisa, Università di Pisa, Scuola Normale Superiore di Pisa, Pisa, Italy; 76 INFN Sezione di Roma, Sapienza Università di Roma, Rome, Italy; 77 INFN Sezione di Torino, Università di Torino, Torino, Italy, Università del Piemonte Orientale, Novara, Italy; 78 INFN Sezione di Trieste, Università di Trieste, Trieste, Italy; 79 Kyungpook National University, Daegu, Korea; 80 Chonnam National University, Institute for Universe and Elementary Particles, Kwangju, Korea; 81 Hanyang University, Seoul, Korea; 82 Korea University, Seoul, Korea; 83 Sejong University, Seoul, Korea; 84 Seoul National University, Seoul, Korea; 85 University of Seoul, Seoul, Korea; 86 Sungkyunkwan University, Suwon, Korea; 87 Vilnius University, Vilnius, Lithuania; 88 National Centre for Particle Physics, Universiti Malaya, Kuala Lumpur, Malaysia; 89 Universidad de Sonora (UNISON), Hermosillo, Mexico; 90 Centro de Investigacion y de Estudios Avanzados del IPN, Mexico City, Mexico; 91 Universidad Iberoamericana, Mexico City, Mexico; 92 Benemerita Universidad Autonoma de Puebla, Puebla, Mexico; 93 Universidad Autónoma de San Luis Potosí, San Luis Potosí, Mexico; 94 University of Auckland, Auckland, New Zealand; 95 University of Canterbury, Christchurch, New Zealand; 96 National Centre for Physics, Quaid-I-Azam University, Islamabad, Pakistan; 97 National Centre for Nuclear Research, Swierk, Poland; 98 Institute of Experimental Physics, Faculty of Physics, University of Warsaw, Warsaw, Poland; 99 Laboratório de Instrumentação e Física Experimental de Partículas, Lisbon, Portugal; 100 Joint Institute for Nuclear Research, Dubna, Russia; 101 Petersburg Nuclear Physics Institute, Gatchina (St. Petersburg), Russia; 102 Institute for Nuclear Research, Moscow, Russia; 103 Institute for Theoretical and Experimental Physics, Moscow, Russia; 104 Moscow Institute of Physics and Technology, Moscow, Russia; 105 National Research Nuclear University ‘Moscow Engineering Physics Institute’ (MEPhI), Moscow, Russia; 106 P.N. Lebedev Physical Institute, Moscow, Russia; 107 Skobeltsyn Institute of Nuclear Physics, Lomonosov Moscow State University, Moscow, Russia; 108 Novosibirsk State University (NSU), Novosibirsk, Russia; 109 Institute for High Energy Physics of National Research Centre ‘Kurchatov Institute’, Protvino, Russia; 110 National Research Tomsk Polytechnic University, Tomsk, Russia; 111 University of Belgrade, Faculty of Physics and Vinca Institute of Nuclear Sciences, Belgrade, Serbia; 112 Centro de Investigaciones Energéticas Medioambientales y Tecnológicas (CIEMAT), Madrid, Spain; 113 Universidad Autónoma de Madrid, Madrid, Spain; 114 Universidad de Oviedo, Oviedo, Spain; 115 Instituto de Física de Cantabria (IFCA), CSIC-Universidad de Cantabria, Santander, Spain; 116 CERN, European Organization for Nuclear Research, Geneva, Switzerland; 117 Paul Scherrer Institut, Villigen, Switzerland; 118 ETH Zurich - Institute for Particle Physics and Astrophysics (IPA), Zurich, Switzerland; 119 Universität Zürich, Zurich, Switzerland; 120 National Central University, Chung-Li, Taiwan; 121 National Taiwan University (NTU), Taipei, Taiwan; 122 Chulalongkorn University, Faculty of Science, Department of Physics, Bangkok, Thailand; 123 Çukurova University, Physics Department, Science and Art Faculty, Adana, Turkey; 124 Middle East Technical University, Physics Department, Ankara, Turkey; 125 Bogazici University, Istanbul, Turkey; 126 Istanbul Technical University, Istanbul, Turkey; 127 Institute for Scintillation Materials of National Academy of Science of Ukraine, Kharkov, Ukraine; 128 National Scientific Center, Kharkov Institute of Physics and Technology, Kharkov, Ukraine; 129 University of Bristol, Bristol, UK; 130 Rutherford Appleton Laboratory, Didcot, UK; 131 Imperial College, London, UK; 132 Brunel University, Uxbridge, UK; 133 Baylor University, Waco, USA; 134 Catholic University of America, Washington, DC USA; 135 The University of Alabama, Tuscaloosa, USA; 136 Boston University, Boston, USA; 137 Brown University, Providence, USA; 138 University of California, Davis, Davis, USA; 139 University of California, Los Angeles, USA; 140 University of California, Riverside, Riverside, USA; 141 University of California, San Diego, La Jolla, USA; 142 University of California, Santa Barbara - Department of Physics, Santa Barbara, USA; 143 California Institute of Technology, Pasadena, USA; 144 Carnegie Mellon University, Pittsburgh, USA; 145 University of Colorado Boulder, Boulder, USA; 146 Cornell University, Ithaca, USA; 147 Fermi National Accelerator Laboratory, Batavia, USA; 148 University of Florida, Gainesville, USA; 149 Florida International University, Miami, USA; 150 Florida State University, Tallahassee, USA; 151 Florida Institute of Technology, Melbourne, USA; 152 University of Illinois at Chicago (UIC), Chicago, USA; 153 The University of Iowa, Iowa City, USA; 154 Johns Hopkins University, Baltimore, USA; 155 The University of Kansas, Lawrence, USA; 156 Kansas State University, Manhattan, USA; 157 Lawrence Livermore National Laboratory, Livermore, USA; 158 University of Maryland, College Park, USA; 159 Massachusetts Institute of Technology, Cambridge, USA; 160 University of Minnesota, Minneapolis, USA; 161 University of Mississippi, Oxford, USA; 162 University of Nebraska-Lincoln, Lincoln, USA; 163 State University of New York at Buffalo, Buffalo, USA; 164 Northeastern University, Boston, USA; 165 Northwestern University, Evanston, USA; 166 University of Notre Dame, Notre Dame, USA; 167 The Ohio State University, Columbus, USA; 168 Princeton University, Princeton, USA; 169 University of Puerto Rico, Mayaguez, USA; 170 Purdue University, West Lafayette, USA; 171 Purdue University Northwest, Hammond, USA; 172 Rice University, Houston, USA; 173 University of Rochester, Rochester, USA; 174 The Rockefeller University, New York, USA; 175 Rutgers, The State University of New Jersey, Piscataway, USA; 176 University of Tennessee, Knoxville, USA; 177 Texas A& M University, College Station, USA; 178 Texas Tech University, Lubbock, USA; 179 Vanderbilt University, Nashville, USA; 180 University of Virginia, Charlottesville, USA; 181 Wayne State University, Detroit, USA; 182 University of Wisconsin-Madison, Madison, WI USA; 183 CERN, 1211 Geneva 23, Switzerland

## Abstract

Exclusive ${{{\uprho _{}^{}} _{}^{}}{{\left( {770}\right) }{}_{}^{}}} ^{0}$ photoproduction is measured for the first time in ultraperipheral pPb collisions at $\sqrt{\smash [b]{s_{_{\mathrm {NN}}}}} = 5.02\,\text {Te}\text {V}$ with the CMS detector. The cross section $\sigma ({\upgamma _{}^{}} \mathrm{p}\rightarrow {{{\uprho _{}^{}} _{}^{}}{{\left( {770}\right) }{}_{}^{}}} ^{0}\mathrm{p})$ is $11.0 \pm 1.4\,\text {(stat)} \pm 1.0\,\text {(syst)}$ $\mu$b at $\langle W_{{\upgamma _{}^{}} \mathrm{p}}\rangle = 92.6\,\text {Ge}\text {V}$ for photon–proton centre-of-mass energies $W_{{\upgamma _{}^{}} \mathrm{p}}$ between 29 and $213\,\text {Ge}\text {V}$. The differential cross section $\mathrm {d}\sigma /\mathrm {d}|t |$ is measured in the interval $0.025< |t | < 1\,\text {Ge}\text {V} ^{2}$ as a function of $W_{{\upgamma _{}^{}} \mathrm{p}}$, where *t* is the squared four-momentum transfer at the proton vertex. The results are compared with previous measurements and theoretical predictions. The measured cross section $\sigma ({\upgamma _{}^{}} \mathrm{p}\rightarrow {{{\uprho _{}^{}} _{}^{}}{{\left( {770}\right) }{}_{}^{}}} ^{0}\mathrm{p})$ has a power-law dependence on the photon–proton centre-of-mass, consistent with electron–proton collision measurements performed at HERA. The $W_{{\upgamma _{}^{}} \mathrm{p}}$ dependence of the exponential slope of the differential cross section $\mathrm {d}\sigma /\mathrm {d}|t |$ is also measured.

## Introduction

Exclusive vector meson (VM) photoproduction, ${\upgamma _{}^{}} \mathrm{p}\rightarrow \mathrm {VM}\mathrm{p}$, has received renewed interest following recent studies of ultraperipheral collisions involving ions and protons at the CERN LHC [1, 2]. In such collisions, photon-induced interactions predominantly occur when the colliding hadrons are separated by a distance larger than the sum of their radii. In this case, one of the hadrons may emit a quasi-real photon that fluctuates into a quark-antiquark pair with the quantum numbers of the photon, which can then turn into a VM upon interacting with the other hadron. The interaction of the VM with the hadron proceeds via the exchange of the vacuum quantum numbers, the so-called pomeron exchange. Proton–lead (pPb) collisions are particularly interesting for studying photon–proton interactions [3, 4] because the large electric charge of the Pb nucleus strongly enhances photon emission. Also, in these events, one can determine the photon direction and hence the photon–proton centre-of-mass energy $W_{{\upgamma _{}^{}} \mathrm{p}}$ unambiguously. This advantage is not present in symmetric colliding systems such as pp interactions. Exclusive VM photoproduction is interesting because the Fourier transform of the *t* distribution, with *t* being the squared four-momentum transfer at the proton vertex, is related to the two-dimensional spatial distribution of the struck partons in the plane transverse to the beam direction. Furthermore, some models suggest that the energy dependence of the integrated cross section and that of the *t* distribution may provide evidence of gluon saturation, as discussed in Refs. [5–10].

By using ultraperipheral pPb collisions at $\sqrt{\smash [b]{s_{_{\mathrm {NN}}}}} = 5.02\,\text {Te}\text {V}$ at the LHC, the ALICE Collaboration has measured the exclusive photoproduction of ${{J/\uppsi _{}^{}}}{{\left( {1S}\right) }{}_{}^{}}$ mesons in the centre-of-mass energy interval $20< W_{{\upgamma _{}^{}} \mathrm{p}} < 700\,\text {Ge}\text {V}$ [11, 12]. The LHCb Collaboration has studied exclusive ${{J/\uppsi _{}^{}}}{{\left( {1S}\right) }{}_{}^{}}$, ${{\uppsi _{}^{}}}{{\left( {2S}\right) }{}_{}^{}}$, and $\Upsilon _{}^{}$ (nS) photoproduction in pp collisions at $\sqrt{s} = 7$ and $8\,\text {Te}\text {V}$ [13, 14]. Exclusive photoproduction of ${{{\uprho _{}^{}} _{}^{}}{{\left( {770}\right) }{}_{}^{}}} ^{0}$ mesons was first studied in fixed-target experiments at $W_{{\upgamma _{}^{}} \mathrm{p}}$ values up to 20$\,\text {Ge}\text {V}$ [15, 16]. Experiments at the HERA electron–proton collider at DESY have studied this process at $W_{{\upgamma _{}^{}} \mathrm{p}}$ values ranging from 50 to 187$\,\text {Ge}\text {V}$, both with quasi-real photons and for photons with larger virtualities [17, 18]. The HERA data have provided clear experimental evidence for the transition from the soft to the hard diffractive regime [19, 20]. More recently, exclusive photoproduction of ${{{\uprho _{}^{}} _{}^{}}{{\left( {770}\right) }{}_{}^{}}} ^{0}$ mesons has been studied by the STAR Collaboration in ultraperipheral AuAu collisions at the BNL RHIC collider [21–23], and by the ALICE Collaboration in PbPb collisions [24]. The cross sections measured by the ALICE and STAR Collaborations in photon-nucleus interactions are 40% lower than both the prediction from the Glauber approach and the corresponding measurements in photon–proton interactions [24, 25]. However, the Glauber approach reproduces the measured cross sections well at lower energies. This is an indication that nuclei do not behave as a collection of independent nucleons at high energies. In the present analysis, exclusive photoproduction of ${{{\uprho _{}^{}} _{}^{}}{{\left( {770}\right) }{}_{}^{}}} ^{0}$ mesons in the ${\uppi _{}^{}} _{}^{+}$ ${\uppi _{}^{}} _{}^{-}$ decay channel in ultraperipheral pPb collisions at $\sqrt{\smash [b]{s_{_{\mathrm {NN}}}}} = 5.02$ $\,\text {Te}\text {V}$ is measured. The cross section is measured as a function of $W_{{\upgamma _{}^{}} \mathrm{p}}$ and *t*. In this paper $|t |$ is defined as the squared transverse momentum of the ${{{\uprho _{}^{}} _{}^{}}{{\left( {770}\right) }{}_{}^{}}} ^0$ meson, $|t | \approx p_{\mathrm {T}} ^{2}$.

This paper is organized as follows. Section 2 describes the experimental apparatus and Sect. 3 the data and simulated Monte Carlo samples. The event selection procedure is illustrated in Sect. 4. Section 5 discusses the background contributions and Sect. 6 the strategy used to extract the signal; the systematic uncertainties are summarized in Sect. 7. The total and differential cross sections are presented in Sect. 8. The results are summarized in Sect. 9.

## The CMS detector

The central feature of the CMS apparatus is a superconducting solenoid of 6$\,\text {m}$ internal diameter, providing a magnetic field of 3.8$\,\text {T}$. Within the solenoid volume are a silicon pixel and strip tracker, a lead tungsten crystal electromagnetic calorimeter (ECAL), and a brass and scintillator hadron calorimeter (HCAL), each composed of a barrel and two endcap sections. The silicon tracker measures charged particles within the range $|\eta |< 2.5$. It consists of 1440 silicon pixel and 15,148 silicon-strip detector modules and is located in the field of the superconducting solenoid. For nonisolated particles of $1< p_{\mathrm {T}} < 10\,\text {Ge}\text {V}$ and $|\eta | < 1.4$, the track resolutions are typically 1.5% in $p_{\mathrm {T}}$ and 25–90 (45–150)$\,\mu \text {m}$ in the transverse (longitudinal) direction [26].

The pseudorapidity coverage for the ECAL and HCAL detectors is $|\eta |<3.0$. The ECAL provides coverage in the pseudorapidity range $|\eta |<1.5$ in the barrel (EB) region and $1.5<|\eta |<3.0$ in the two endcap (EE) regions. The HCAL provides coverage for $|\eta |<1.3$ in the barrel (HB) region and $1.3<|\eta |< 3.0$ in the two endcap (HE) regions. The hadron forward (HF) calorimeters ($3.0<|\eta | < 5.2$) complement the coverage provided by the barrel and endcap detectors. The zero-degree calorimeters (ZDCs) are two Čerenkov calorimeters composed of alternating layers of tungsten and quartz fibers that cover the region $|\eta |>8.3$. Both the HF and ZDC detectors are divided into two halves, one covering positive pseudorapidities, the other negative, and referred to as HF+ and ZDC+ (and HF- and ZDC-), respectively. Another calorimeter, CASTOR, also a Čerenkov sampling calorimeter, consists of quartz and tungsten plates and is located only at negative pseudorapidities with coverage of $-6.6<\eta <-5.2$.

A more detailed description of the CMS detector, together with the definition of the coordinate system used and the relevant kinematic variables, can be found in Ref. [27].

## Data and Monte Carlo simulation

This analysis uses data from pPb collisions at $\sqrt{\smash [b]{s_{_{\mathrm {NN}}}}} = 5.02\,\text {Te}\text {V}$ collected with the CMS detector in February 2013. The beam energies are 4$\,\text {Te}\text {V}$ for the protons and 1.58$\,\text {Te}\text {V}$ per nucleon for the lead nuclei. The integrated luminosity is ${\mathcal {L}} = 7.4\,\mu \text {b}^{-1}$ for the pPb data set (protons circulating in the negative *z* direction) and ${\mathcal {L}} = 9.6\,\mu \text {b}^{-1}$ for the Pbp data set (protons circulating in the positive *z* direction). Since the events are asymmetric in rapidity, the pPb and Pbp samples are merged after changing the sign of the rapidity in the Pbp sample.

The starlight (version 2.2.0) Monte Carlo (MC) event generator [28] is used to simulate exclusive ${{{\uprho _{}^{}} _{}^{}}{{\left( {770}\right) }{}_{}^{}}} ^{0}$ photoproduction followed by the ${{{\uprho _{}^{}} _{}^{}}{{\left( {770}\right) }{}_{}^{}}} ^{0}\rightarrow {{\uppi _{}^{}} _{}^{+}} {{\uppi _{}^{}} _{}^{-}}$ decay. The starlight generator models two-photon and photon-hadron interactions at ultrarelativistic energies. Two processes contribute to the exclusive ${{\uppi _{}^{}} _{}^{+}} {{\uppi _{}^{}} _{}^{-}}$ channel: resonant ${{{\uprho _{}^{}} _{}^{}}{{\left( {770}\right) }{}_{}^{}}} ^{0}\rightarrow {\uppi _{}^{}} ^{+}{\uppi _{}^{}} ^{-}$ production, and nonresonant ${{\uppi _{}^{}} _{}^{+}} {{\uppi _{}^{}} _{}^{-}}$ production, including the interference term. Both processes are generated in order to calculate the signal acceptance and efficiency, and to extract the corrected signal yield. starlight is also used to generate exclusive ${{\uprho _{}^{}} _{}^{}}{{\left( {1700}\right) }{}_{}^{}}$ events. The pPb and Pbp samples are produced separately. The events are passed through a detailed Geant4 [29] simulation of the CMS detector in order to model the detector response, and are reconstructed with the same software used for the data.

## Event selection

Table 1 presents the number of events after each selection requirement is applied. Events were selected online [30] by requiring the simultaneous presence of the two beams at the interaction point, as measured by the beam monitor timing system, in conjunction with at least one track in the pixel tracker. Offline, events are discarded if they have an energy deposit in any of the HF towers above the noise threshold of 3$\,\text {Ge}\text {V}$. Events are also required to have exactly two tracks that pass the selection criteria defined in Ref. [31], and to be associated with a single vertex located within 15$\,\text {cm}$ of the nominal interaction point along the beam direction. The pion mass is assigned to each track. In order to minimize the effect of the uncertainty in the low-$p_{\mathrm {T}}$ track efficiency, one of the tracks should have a $p_{\mathrm {T}}$ larger than 0.4$\,\text {Ge}\text {V}$, and the other larger than 0.2$\,\text {Ge}\text {V}$. Both tracks are selected in the interval $|\eta | < 2.0$. The rapidity of the ${{\uppi _{}^{}} _{}^{+}} {{\uppi _{}^{}} _{}^{-}}$ system is required to be in the interval $|y_{{{\uppi _{}^{}} _{}^{+}} {{\uppi _{}^{}} _{}^{-}}} |<2.0$. To reject the photoproduction of ${{{\uprho _{}^{}} _{}^{}}{{\left( {770}\right) }{}_{}^{}}} ^{0}$ mesons from ${\upgamma _{}^{}} \mathrm {Pb}$ interactions where the proton radiates a quasi-real photon, the $p_{\mathrm {T}}$ of the ${{\uppi _{}^{}} _{}^{+}} {{\uppi _{}^{}} _{}^{-}}$ system is required to be larger than 0.15$\,\text {Ge}\text {V}$ (as discussed in Sect. 5).

A sizable background contribution comes from proton dissociative events, ${\upgamma _{}^{}} \mathrm{p}\rightarrow {{{\uprho _{}^{}} _{}^{}}{{\left( {770}\right) }{}_{}^{}}} ^{0}\mathrm{p}^{*}$, where $\mathrm{p}^{*}$ indicates a low-mass hadronic state. In these events the scattered proton is excited and then dissociates. The ${{{\uprho _{}^{}} _{}^{}}{{\left( {770}\right) }{}_{}^{}}} ^{0}$ is measured in the central region, whereas the low-mass state usually escapes undetected. To suppress this contribution, events with activity above noise thresholds in the CASTOR, HE, HF, and ZDC detectors are rejected. The signal-to-noise ratio in $\hbox {ZDC}^{+}$ is better than in $\hbox {ZDC}^{-}$ because of differences in radiation damage to the two detectors. For this reason, the ZDC energy thresholds shown in Table 1 are asymmetric. CASTOR is used for only the pPb sample because of its location, as discussed in Sect. 2. The final selection requires the two tracks to have opposite charges. A total of 20,060 opposite-sign pair events and 1514 same-sign pair events are selected in this analysis.

**Table 1:** Integrated luminosity and number of events after each of the selection requirements for the two data samples. The leading tower is the tower with the largest energy deposition in the calorimeter

Selection	Number of selected events	
	pPb	Pbp
Integrated luminosity	7.4 $\,\mu \text {b}^{-1}$	9.6 $\,\mu \text {b}^{-1}$
Leading HF tower $< 3.0$ $\,\text {Ge}\text {V}$	52,508	66,278
Exactly two tracks	17,771	21,583
Track purity [31]	16,085	20,278
$|\eta _\text {track} |<2.0$,	12,707	16,037
$p_{\mathrm {T}} ^\text {leading} >0.4$ $\,\text {Ge}\text {V}$, $p_{\mathrm {T}} ^{\text {subleading}}>0.2$ $\,\text {Ge}\text {V}$	12,364	15,572
$|z_{\mathrm {vertex}} | < 15$ $\,\text {cm}$	11,924	15,052
Leading HE tower $< 1.95$ $\,\text {Ge}\text {V}$	11,563	14,643
CASTOR energy $< 9$ $\,\text {Ge}\text {V}$	9405	–
$\hbox {ZDC}^{{+}}$ energy $< 500$ $\,\text {Ge}\text {V}$	–	12 475
$\hbox {ZDC}^{{-}}$ energy $< 2000$ $\,\text {Ge}\text {V}$	9099	–
Opposite-sign pairs	8507	11,553
Same-sign pairs	592	922

## Background

The main background sources are listed below.

**Fig. 1:**
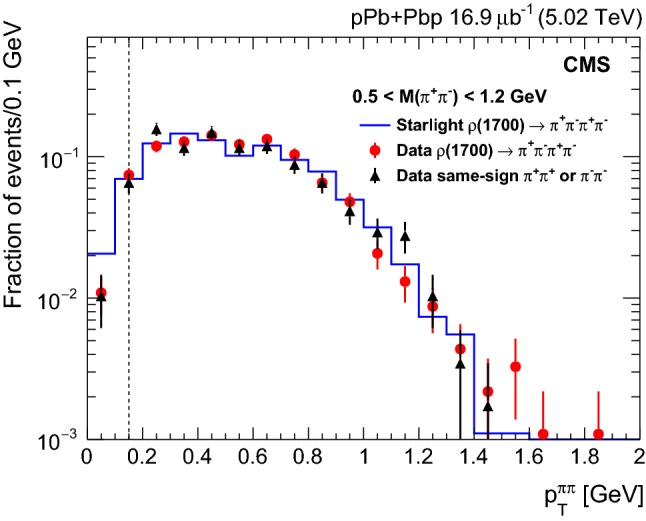
Comparison between the $p_{\mathrm {T}} ^{{{\uppi _{}^{}} _{}^{+}} {{\uppi _{}^{}} _{}^{-}}}$ distributions of the reconstructed ${{{\uprho _{}^{}} _{}^{}}{{\left( {1700}\right) }{}_{}^{}}}$ mesons in the data (full symbols) and the starlight simulation (histogram) when only two oppositely charged pions are selected. The triangles correspond to same-sign two-track events (either ${{\uppi _{}^{}} _{}^{+}} {{\uppi _{}^{}} _{}^{+}}$ or ${{\uppi _{}^{}} _{}^{-}} {{\uppi _{}^{}} _{}^{-}}$) in the data; they mostly come from ${{{\uprho _{}^{}} _{}^{}}{{\left( {1700}\right) }{}_{}^{}}}$ decays with two undetected pions. The integrals of all three distributions are normalized to unity. Vertical bars correspond to the statistical uncertainties. The region to the left of the dashed vertical line is not included in the analysis (see Sect. 5 for details)

- *Nonresonant* ${{\uppi _{}^{}} _{}^{+}} {{\uppi _{}^{}} _{}^{-}}$ *production*. This contributes mainly through an interference term. It is included when fitting the invariant mass distribution, as discussed in Sect. 6.
- *Exclusive photoproduction of* ${{{\upomega _{}^{}}}{{\left( {783}\right) }{}_{}^{}}}$ *and* ${{{\upphi _{}^{}}}{{\left( {1020}\right) }{}_{}^{}}}$ *mesons*. Contamination from the decay ${{{\upphi _{}^{}}}{{\left( {1020}\right) }{}_{}^{}}} \rightarrow \hbox {K}^{+}\hbox {K}^{-}$ is removed by assigning the kaon mass to the tracks and rejecting events with invariant mass values of the $\hbox {K}^{+}\hbox {K}^{-}$ system larger than 1.04$\,\text {Ge}\text {V}$. In addition, contamination is expected from the ${{{\upomega _{}^{}}}{{\left( {783}\right) }{}_{}^{}}} \rightarrow {{\uppi _{}^{}} _{}^{+}} {{\uppi _{}^{}} _{}^{-}} {{\uppi _{}^{}} _{}^{0}}$ and ${{{\upphi _{}^{}}}{{\left( {1020}\right) }{}_{}^{}}} \rightarrow {{\uppi _{}^{}} _{}^{+}} {{\uppi _{}^{}} _{}^{-}} {{\uppi _{}^{}} _{}^{0}}$ decays when the photons from the ${\uppi _{}^{}} _{}^{0}$  decay are undetected. Although the ${{\uppi _{}^{}} _{}^{+}} {{\uppi _{}^{}} _{}^{-}}$ invariant mass in these cases is mostly below the ${{\uprho _{}^{}} _{}^{}}{{\left( {770}\right) }{}_{}^{}}$  mass, the rate of ${{{\upomega _{}^{}}}{{\left( {783}\right) }{}_{}^{}}}$ and ${{{\upphi _{}^{}}}{{\left( {1020}\right) }{}_{}^{}}}$ meson production increases with $|t |$. As observed in this analysis and at HERA [32], undetected photons lead to an overestimate of the $p_{\mathrm {T}}$ imbalance in the event, mimicking large $|t |$ events. Since these processes cannot be modeled by starlight, their contribution is estimated from the fits of the unfolded invariant mass distributions described in Sect. 6. The ${{{\upomega _{}^{}}}{{\left( {783}\right) }{}_{}^{}}} \rightarrow {{\uppi _{}^{}} _{}^{+}} {{\uppi _{}^{}} _{}^{-}}$ amplitude is small, but is clearly visible through its interference with the ${{{\uprho _{}^{}} _{}^{}}{{\left( {770}\right) }{}_{}^{}}} ^{0}$, which produces the small kink in the invariant mass spectrum near 800$\,\text {Me}\text {V}$. This contribution is included in the invariant mass fit, as discussed in Sect. 6.
- *Exclusive photoproduction of* ${{{\uprho _{}^{}} _{}^{}}{{\left( {1700}\right) }{}_{}^{}}}$ *mesons*.^1^ The ${{{\uprho _{}^{}} _{}^{}}{{\left( {1700}\right) }{}_{}^{}}}$ decays mostly into a ${{{\uprho _{}^{}} _{}^{}}{{\left( {770}\right) }{}_{}^{}}} ^{0}$ meson and a pion pair, leading to final states with four charged pions, or with two charged pions and two neutral pions. The ${{{\uprho _{}^{}} _{}^{}}{{\left( {1700}\right) }{}_{}^{}}} \rightarrow {{\uppi _{}^{}} _{}^{+}} {{\uppi _{}^{}} _{}^{-}} {{\uppi _{}^{}} _{}^{+}} {{\uppi _{}^{}} _{}^{-}}$ decay may also result in opposite-sign events when only two opposite-sign pions are detected because of the limited rapidity coverage of the detector. Such events will appear to have a $p_{\mathrm {T}}$ imbalance, causing them to be incorrectly identified as large $|t |~{{{\uprho _{}^{}} _{}^{}}{{\left( {770}\right) }{}_{}^{}}} ^0$ events, thereby resulting in a distortion of the $|t |$ distribution. To validate the use of starlight for ${{{\uprho _{}^{}} _{}^{}}{{\left( {1700}\right) }{}_{}^{}}}$ photoproduction, exclusive ${{\uppi _{}^{}} _{}^{+}} {{\uppi _{}^{}} _{}^{-}} {{\uppi _{}^{}} _{}^{+}} {{\uppi _{}^{}} _{}^{-}}$ events are selected in the data. The data sample and the starlight simulation for ${{{\uprho _{}^{}} _{}^{}}{{\left( {1700}\right) }{}_{}^{}}}$ exclusive photoproduction are studied by applying the same selection criteria as for the ${{{\uprho _{}^{}} _{}^{}}{{\left( {770}\right) }{}_{}^{}}} ^{0}$, except that on the number of tracks. Figure 1 shows a comparison of the $p_{\mathrm {T}} ^{{{\uppi _{}^{}} _{}^{+}} {{\uppi _{}^{}} _{}^{-}}}$ distributions of the reconstructed ${{{\uprho _{}^{}} _{}^{}}{{\left( {1700}\right) }{}_{}^{}}}$ mesons in the four pion event samples obtained from the data and the starlight simulation. All combinations of two oppositely charged pions are plotted in Fig. 1 if they have an invariant mass $0.5< M_{{{\uppi _{}^{}} _{}^{+}} {{\uppi _{}^{}} _{}^{-}}} < 1.2\,\text {Ge}\text {V}$. In addition, the distribution of the same-sign events in the data is shown; they come mostly from ${{{\uprho _{}^{}} _{}^{}}{{\left( {1700}\right) }{}_{}^{}}}$ decays with two missing pions. Figure 1 shows that the data and the starlight results are in agreement, lending confidence to the performance of this generator. These distributions provide a template for the $p_{\mathrm {T}} ^{{{\uppi _{}^{}} _{}^{+}} {{\uppi _{}^{}} _{}^{-}}}$ distribution of the ${{{\uprho _{}^{}} _{}^{}}{{\left( {1700}\right) }{}_{}^{}}}$ background used to estimate its contribution, as described in Sect. 6.
- *Proton dissociative* ${{{\uprho _{}^{}} _{}^{}}{{\left( {770}\right) }{}_{}^{}}} ^{0}$ *photoproduction*. This contribution is suppressed by rejecting events with activity in the CASTOR, HE, HF, and ZDC detectors. In order to determine the residual contribution, a sample of dissociative events is selected by requiring activity in at least one of the forward detectors (CASTOR, HF, or ZDC). This sample provides a template for the $p_{\mathrm {T}} ^{{{\uppi _{}^{}} _{}^{+}} {{\uppi _{}^{}} _{}^{-}}}$ distribution of the dissociative events, under the assumption that the $p_{\mathrm {T}} ^{{{\uppi _{}^{}} _{}^{+}} {{\uppi _{}^{}} _{}^{-}}}$ distribution is independent of the mass of the dissociative system (the more forward the detector, the smaller the masses to which it is sensitive). Finally, this template is used to estimate the remaining dissociative background contributions, as discussed in Sect. 6.
- *Double pomeron exchange processes and photoproduction processes from* ${\upgamma _{}^{}} \mathrm {Pb}$ *interactions*. Since the strong force has short range, only the nucleons on the surface of the nucleus may contribute to double pomeron exchange interactions; the corresponding cross section is therefore negligible [35]. For coherent processes in ${\upgamma _{}^{}} \mathrm {Pb}$ interactions, the size of the lead ion restricts the mean $p_{\mathrm {T}}$ of the VM to be about $60\,\text {Me}\text {V}$, corresponding to a de Broglie wavelength of the order of the nucleus size. Taking into account the detector resolution, all coherent ${{{\uprho _{}^{}} _{}^{}}{{\left( {770}\right) }{}_{}^{}}} ^{0}$ events have $p_{\mathrm {T}}$ less than $0.15\,\text {Ge}\text {V}$. Thus, events from ${\upgamma _{}^{}} \mathrm {Pb}$ interactions contribute to the lowest $|t |$ region, which is not included in this analysis.

## Signal extraction

The extraction of the signal is carried out in two steps. First, the proton dissociative and the ${{{\uprho _{}^{}} _{}^{}}{{\left( {1700}\right) }{}_{}^{}}}$ contributions are estimated by performing a fit to the data as a function of $p_{\mathrm {T}} ^{{{\uppi _{}^{}} _{}^{+}} {{\uppi _{}^{}} _{}^{-}}}$. This method relies on the fact that exclusive ${{{\uprho _{}^{}} _{}^{}}{{\left( {770}\right) }{}_{}^{}}} ^{0}$ events contribute mainly to the low-$p_{\mathrm {T}} ^{{{\uppi _{}^{}} _{}^{+}} {{\uppi _{}^{}} _{}^{-}}}$ region ($p_{\mathrm {T}} ^{{{\uppi _{}^{}} _{}^{+}} {{\uppi _{}^{}} _{}^{-}}}<0.7\,\text {Ge}\text {V}$), whereas nonexclusive events dominate the high-$p_{\mathrm {T}} ^{{{\uppi _{}^{}} _{}^{+}} {{\uppi _{}^{}} _{}^{-}}}$ region ($p_{\mathrm {T}} ^{{{\uppi _{}^{}} _{}^{+}} {{\uppi _{}^{}} _{}^{-}}}>1.2\,\text {Ge}\text {V}$), and the ${{{\uprho _{}^{}} _{}^{}}{{\left( {1700}\right) }{}_{}^{}}}$ contribution is mostly at intermediate $p_{\mathrm {T}} ^{{{\uppi _{}^{}} _{}^{+}} {{\uppi _{}^{}} _{}^{-}}}$ values ($0.7<p_{\mathrm {T}} ^{{{\uppi _{}^{}} _{}^{+}} {{\uppi _{}^{}} _{}^{-}}}<1.2\,\text {Ge}\text {V}$). This makes the identification of the proton dissociative and the ${{{\uprho _{}^{}} _{}^{}}{{\left( {1700}\right) }{}_{}^{}}}$ contributions robust. Second, the yield of exclusive ${{{\uprho _{}^{}} _{}^{}}{{\left( {770}\right) }{}_{}^{}}} ^{0}$ candidates is extracted by performing a fit to the unfolded invariant mass distribution. Since the events from exclusive ${{{\uprho _{}^{}} _{}^{}}{{\left( {1700}\right) }{}_{}^{}}}$ production have a different invariant mass distribution from the signal events, they are subtracted before correcting the data for acceptance and efficiency. Conversely, the proton dissociative background has the same invariant mass and angular distributions as the signal, and its effect is corrected after unfolding by scaling the observed yields according to the fit performed in the first step.

To extract the normalizations of the proton dissociative and the ${{{\uprho _{}^{}} _{}^{}}{{\left( {1700}\right) }{}_{}^{}}}$ backgrounds, an unbinned maximum likelihood fit is performed to the data as a function of $p_{\mathrm {T}} ^{{{\uppi _{}^{}} _{}^{+}} {{\uppi _{}^{}} _{}^{-}}}$ in the rapidity interval $|y_{{{\uppi _{}^{}} _{}^{+}} {{\uppi _{}^{}} _{}^{-}}} | < 2$. The sum of the following distributions is fitted to the data at the reconstructed level: the signal distribution and the ${{\uppi _{}^{}} _{}^{+}} {{\uppi _{}^{}} _{}^{-}}$ continuum, as simulated by starlight, the distribution of the proton dissociative background, which is extracted from the data control sample, and the ${{{\uprho _{}^{}} _{}^{}}{{\left( {1700}\right) }{}_{}^{}}}$ fitting template, which is simulated using starlight. The normalization of each of these components is determined from the fit. The signal $p_{\mathrm {T}} ^{{{\uppi _{}^{}} _{}^{+}} {{\uppi _{}^{}} _{}^{-}}}$ distribution generated by starlight is reweighted to describe the data using the theory-inspired expression $\mathrm {e}^{- b|t |}$ [15]. The initial *b* value of starlight is $12\,\text {Ge}\text {V} ^{-2}$ and the reweighted *b* is $13.1^{+0.4}_{-0.3}\,\text {(stat)} \,\text {Ge}\text {V} ^{-2}$.

**Fig. 2:**
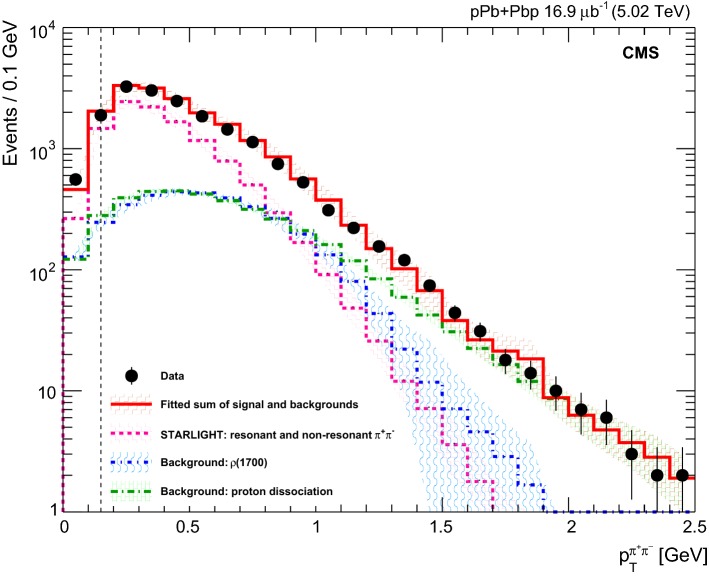
The measured distribution of the reconstructed ${{\uppi _{}^{}} _{}^{+}} {{\uppi _{}^{}} _{}^{-}}$ transverse momentum (full circles) together with the fitted sum of signal and backgrounds described in the text (red solid histogram). The starlight direct ${{\uppi _{}^{}} _{}^{+}} {{\uppi _{}^{}} _{}^{-}}$ contribution (pink dotted histogram), the ${{{\uprho _{}^{}} _{}^{}}{{\left( {1700}\right) }{}_{}^{}}}$ background (blue dotted-short-dashed histogram), and the proton-dissociative contribution (green dotted-long-dashed histogram) are also shown. The shaded areas represent the systematic uncertainties. The region to the left of the dashed vertical line is not included in the analysis (see Sect. 5 for details)

The result of the fit of the $p_{\mathrm {T}} ^{{{\uppi _{}^{}} _{}^{+}} {{\uppi _{}^{}} _{}^{-}}}$ distributions is shown in Fig. 2, including the systematic uncertainties associated with the fitting procedure that are discussed in Sect. 7. The resulting residual proton-dissociative and ${{{\uprho _{}^{}} _{}^{}}{{\left( {1700}\right) }{}_{}^{}}}$ contributions, over the whole rapidity interval, are $18\pm 2\%$ $\,\text {(stat)}$ and $20\pm 2\%\,\text {(stat)}$, respectively. Similar fractions of proton dissociative and ${{{\uprho _{}^{}} _{}^{}}{{\left( {1700}\right) }{}_{}^{}}}$ contributions are obtained in the four rapidity intervals used in the differential cross section measurement as a function of rapidity. This is consistent with the small energy dependence of these processes in the energy range of this analysis. As seen in Fig. 2, the signal and both background contributions are of the same order of magnitude around $p_{\mathrm {T}} ^{{{\uppi _{}^{}} _{}^{+}} {{\uppi _{}^{}} _{}^{-}}}=1\,\text {Ge}\text {V}$, corresponding to a signal-to-background ratio of about 30%. For this reason, only the region $|t | < 1$ $\text {Ge}\text {V} ^{2}$ is used in this measurement.

The ${{{\uprho _{}^{}} _{}^{}}{{\left( {1700}\right) }{}_{}^{}}}$ background is subtracted in bins of invariant mass using the normalization obtained from the $p_{\mathrm {T}} ^{{{\uppi _{}^{}} _{}^{+}} {{\uppi _{}^{}} _{}^{-}}}$ fitting templates. The invariant mass distribution is then unfolded using the iterative D’Agostini method [36], which is regularized by four iterations. In particular, the Bayesian iterative unfolding technique is used, as implemented in the  roounfold package [37]. This procedure leads to corrections for experimental effects including possible data migration between bins. The response matrix is obtained from starlight. The average of the combined acceptance and efficiency is 0.13 and is almost independent of both $p_{\mathrm {T}}$ and $\eta$, whereas it is sensitive to the invariant mass.

The invariant mass shape of the ${{{\uprho _{}^{}} _{}^{}}{{\left( {770}\right) }{}_{}^{}}} ^{0}$ in photoproduction deviates from that of a pure Breit–Wigner resonance [38]. Several parameterizations of the shape exist. One of the most often used is the Söding formula [39], where a continuum amplitude *B* is added to a Breit–Wigner distribution. Following the recent results by the STAR Collaboration [23] and the earlier ones by the DESY-MIT Collaboration [40], a further relativistic Breit–Wigner component is added to account for ${{{\upomega _{}^{}}}{{\left( {783}\right) }{}_{}^{}}}$ photoproduction, followed by the decay ${{{\upomega _{}^{}}}{{\left( {783}\right) }{}_{}^{}}} \rightarrow {{\uppi _{}^{}} _{}^{+}} {{\uppi _{}^{}} _{}^{-}}$. This leads to the following fitting function:

$$\begin{aligned}&\frac{\mathrm {d}N_{{{\uppi _{}^{}} _{}^{+}} {{\uppi _{}^{}} _{}^{-}}}}{\mathrm {d}M_{{{\uppi _{}^{}} _{}^{+}} {{\uppi _{}^{}} _{}^{-}}}} = \left| A\frac{\sqrt{M_{{{\uppi _{}^{}} _{}^{+}} {{\uppi _{}^{}} _{}^{-}}}M_{{{{\uprho _{}^{}} _{}^{}}{{\left( {770}\right) }{}_{}^{}}}}\varGamma _{{{{\uprho _{}^{}} _{}^{}}{{\left( {770}\right) }{}_{}^{}}}}}}{M_{{{\uppi _{}^{}} _{}^{+}} {{\uppi _{}^{}} _{}^{-}}}^{2} - M_{{{{\uprho _{}^{}} _{}^{}}{{\left( {770}\right) }{}_{}^{}}} ^{0}}^{2} + iM_{{{{\uprho _{}^{}} _{}^{}}{{\left( {770}\right) }{}_{}^{}}} ^{0}}\varGamma _{{{{\uprho _{}^{}} _{}^{}}{{\left( {770}\right) }{}_{}^{}}}}}\right. \\&\left. \quad + B + C\mathrm {e}^{i\phi _\omega }\frac{\sqrt{M_{{{\uppi _{}^{}} _{}^{+}} {{\uppi _{}^{}} _{}^{-}}}M_{{{{\upomega _{}^{}}}{{\left( {783}\right) }{}_{}^{}}}}\varGamma _{{{{\upomega _{}^{}}}{{\left( {783}\right) }{}_{}^{}}} \rightarrow {\uppi _{}^{}} {\uppi _{}^{}}}}}{M_{{{\uppi _{}^{}} _{}^{+}} {{\uppi _{}^{}} _{}^{-}}}^{2} - M_{{{{\upomega _{}^{}}}{{\left( {783}\right) }{}_{}^{}}}}^{2} + iM_{{{{\upomega _{}^{}}}{{\left( {783}\right) }{}_{}^{}}} ^{0}}\varGamma _{{{{\upomega _{}^{}}}{{\left( {783}\right) }{}_{}^{}}}}} \right| ^{2}. \end{aligned}$$

Here *A* is the amplitude of the ${{{\uprho _{}^{}} _{}^{}}{{\left( {770}\right) }{}_{}^{}}} ^{0}$ Breit–Wigner function, *B* is the amplitude of the direct nonresonant ${{\uppi _{}^{}} _{}^{+}} {{\uppi _{}^{}} _{}^{-}}$ production, *C* is the amplitude of the ${{\upomega _{}^{}}}{{\left( {783}\right) }{}_{}^{}}$ contribution, and the mass-dependent widths are given by

$$\begin{aligned} \varGamma _{{{{\uprho _{}^{}} _{}^{}}{{\left( {770}\right) }{}_{}^{}}}} = \varGamma _{0}\frac{M_{{{{\uprho _{}^{}} _{}^{}}{{\left( {770}\right) }{}_{}^{}}} ^{0}}}{M_{{{\uppi _{}^{}} _{}^{+}} {{\uppi _{}^{}} _{}^{-}}}} \left[ \frac{M_{{{\uppi _{}^{}} _{}^{+}} {{\uppi _{}^{}} _{}^{-}}}^{2} - 4m_{{{\uppi _{}^{}} _{}^{\pm }}}^{2}}{M_{{{{\uprho _{}^{}} _{}^{}}{{\left( {770}\right) }{}_{}^{}}} ^{0}}^{2} - 4m_{{{\uppi _{}^{}} _{}^{\pm }}}^{2}} \right] ^{\frac{3}{2}}, \end{aligned}$$

and

$$\begin{aligned} \varGamma _{{{{\upomega _{}^{}}}{{\left( {783}\right) }{}_{}^{}}}} = \varGamma _{0}\frac{M_{{{{\upomega _{}^{}}}{{\left( {783}\right) }{}_{}^{}}}}}{M_{{{\uppi _{}^{}} _{}^{+}} {{\uppi _{}^{}} _{}^{-}}}} \left[ \frac{M_{{{\uppi _{}^{}} _{}^{+}} {{\uppi _{}^{}} _{}^{-}}}^{2} - 9m_{{{\uppi _{}^{}} _{}^{\pm }}}^{2}}{M_{{{{\upomega _{}^{}}}{{\left( {783}\right) }{}_{}^{}}}}^{2} - 9m_{{{\uppi _{}^{}} _{}^{\pm }}}^{2}} \right] ^{\frac{3}{2}}, \end{aligned}$$

where $\varGamma _{0}$ is the pole width for each meson and $m_{{{\uppi _{}^{}} _{}^{\pm }}}$ is the charged pion mass. Since the branching fraction (${\mathcal {B}}$) for ${{{\upomega _{}^{}}}{{\left( {783}\right) }{}_{}^{}}} \rightarrow {{\uppi _{}^{}} _{}^{+}} {{\uppi _{}^{}} _{}^{-}}$ is small, only the first order term in the ${{{\upomega _{}^{}}}{{\left( {783}\right) }{}_{}^{}}}-{{{\uprho _{}^{}} _{}^{}}{{\left( {770}\right) }{}_{}^{}}} ^{0}$ mass mixing theory is considered [40], leading to

$$\begin{aligned}&\varGamma _{{{{\upomega _{}^{}}}{{\left( {783}\right) }{}_{}^{}}} \rightarrow {\uppi _{}^{}} {\uppi _{}^{}}} \\&\quad ={\mathcal {B}}({{{\upomega _{}^{}}}{{\left( {783}\right) }{}_{}^{}}} \rightarrow {\uppi _{}^{}} {\uppi _{}^{}})\varGamma _{0}\frac{M_{{{{\upomega _{}^{}}}{{\left( {783}\right) }{}_{}^{}}}}}{M_{{{\uppi _{}^{}} _{}^{+}} {{\uppi _{}^{}} _{}^{-}}}} \left[ \frac{M_{{{\uppi _{}^{}} _{}^{+}} {{\uppi _{}^{}} _{}^{-}}}^{2} - 4m_{{{\uppi _{}^{}} _{}^{\pm }}}^{2}}{M_{{{{\upomega _{}^{}}}{{\left( {783}\right) }{}_{}^{}}}}^{2} - 4m_{{{\uppi _{}^{}} _{}^{\pm }}}^{2}} \right] ^{\frac{3}{2}}, \end{aligned}$$

with ${\mathcal {B}}({{{\upomega _{}^{}}}{{\left( {783}\right) }{}_{}^{}}} \rightarrow {\uppi _{}^{}} {\uppi _{}^{}})=0.0153^{+0.0011}_{-0.0013}$ [33]. The H1 and ZEUS measurements did not include the ${{{\upomega _{}^{}}}{{\left( {783}\right) }{}_{}^{}}}-{{{\uprho _{}^{}} _{}^{}}{{\left( {770}\right) }{}_{}^{}}} ^{0}$ interference component, although the ZEUS data seem to indicate its effect in the mass spectrum near 800$\,\text {Me}\text {V}$ [17].

**Fig. 3:**
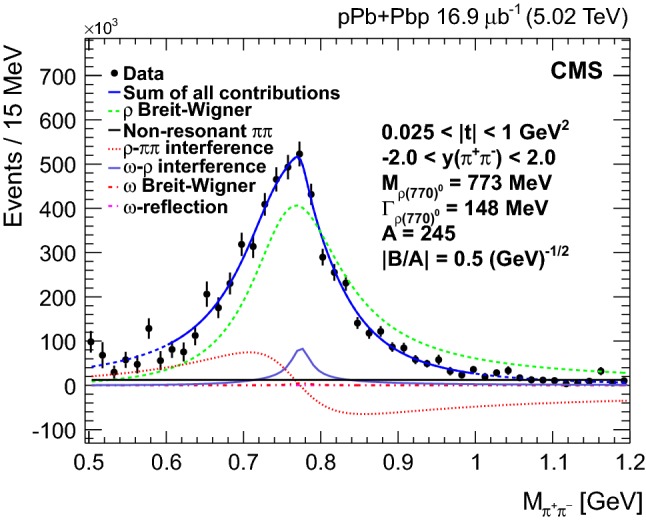
Unfolded ${{\uppi _{}^{}} _{}^{+}} {{\uppi _{}^{}} _{}^{-}}$ invariant mass distribution in the pion pair rapidity interval $|y_{{{\uppi _{}^{}} _{}^{+}} {{\uppi _{}^{}} _{}^{-}}} | < 2.0$ (full circles) fitted with the modified Söding model. The results of the fit are also given (see text for details). The green dashed line indicates resonant ${{{\uprho _{}^{}} _{}^{}}{{\left( {770}\right) }{}_{}^{}}} ^{0}$ production, the red dotted line the interference term, the black dash-dotted line the non-resonant contribution, the dark blue dashed line the interference between ${{{\uprho _{}^{}} _{}^{}}{{\left( {770}\right) }{}_{}^{}}} ^{0}$ and ${{{\upomega _{}^{}}}{{\left( {783}\right) }{}_{}^{}}}$, and the blue solid line represents the sum of all these contributions

**Fig. 4:**
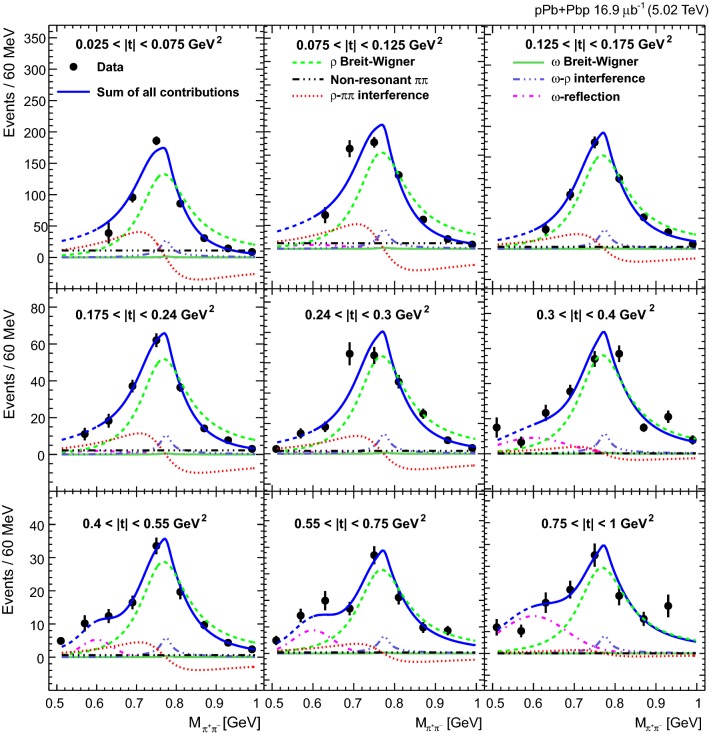
Unfolded ${{\uppi _{}^{}} _{}^{+}} {{\uppi _{}^{}} _{}^{-}}$ invariant mass distributions in the pion pair rapidity interval $|y_{{{\uppi _{}^{}} _{}^{+}} {{\uppi _{}^{}} _{}^{-}}} | < 2.0$ (full circles) fitted with the Söding model in different $|t |$ bins. The green dashed lines indicate resonant ${{{\uprho _{}^{}} _{}^{}}{{\left( {770}\right) }{}_{}^{}}} ^{0}$ production, the red dotted lines the interference term, the magenta dash-dotted lines correspond to the background from ${{{\upomega _{}^{}}}{{\left( {783}\right) }{}_{}^{}}} \rightarrow {{\uppi _{}^{}} _{}^{0}} {{\uppi _{}^{}} _{}^{+}} {{\uppi _{}^{}} _{}^{-}}$, the black dash-dotted lines to the nonresonant contribution, the dark blue dashed line to the interference between ${{{\uprho _{}^{}} _{}^{}}{{\left( {770}\right) }{}_{}^{}}} ^{0}$ and ${{{\upomega _{}^{}}}{{\left( {783}\right) }{}_{}^{}}}$, and the blue solid lines represent the sum of all these contributions

Figure 3 shows the fit of the unfolded distribution with the modifed Söding model. A least squares fit is performed for the interval $0.6< M_{{{\uppi _{}^{}} _{}^{+}} {{\uppi _{}^{}} _{}^{-}}} < 1.1\,\text {Ge}\text {V}$, with the quantities $M_{{{{\uprho _{}^{}} _{}^{}}{{\left( {770}\right) }{}_{}^{}}} ^{0}}$, $M_{{{{\upomega _{}^{}}}{{\left( {783}\right) }{}_{}^{}}}}$, $\varGamma _{{{{\uprho _{}^{}} _{}^{}}{{\left( {770}\right) }{}_{}^{}}} ^{0}}$, $\varGamma _{{{{\upomega _{}^{}}}{{\left( {783}\right) }{}_{}^{}}}}$, *A*, *B*, *C*, and $\phi _{{{\upomega _{}^{}}}{{\left( {783}\right) }{}_{}^{}}}$ treated as free parameters. This model includes the interference between resonant ${{{\uprho _{}^{}} _{}^{}}{{\left( {770}\right) }{}_{}^{}}} ^{0}$ and direct ${\uppi _{}^{}} ^{+}{\uppi _{}^{}} ^{-}$ production, as well as between ${{{\uprho _{}^{}} _{}^{}}{{\left( {770}\right) }{}_{}^{}}} ^{0}$ and ${{{\upomega _{}^{}}}{{\left( {783}\right) }{}_{}^{}}}$ production. To correct for the ${{\upomega _{}^{}}}{{\left( {783}\right) }{}_{}^{}}$  reflection in the ${{\uppi _{}^{}} _{}^{+}} {{\uppi _{}^{}} _{}^{-}}$ mass spectrum, a Gaussian function peaking around 500$\,\text {Me}\text {V}$ [18] is added as a further component of the invariant mass fit. This is only visible at high $|t |$ values, as shown in Fig. 4. The fit yields $M_{{{{\uprho _{}^{}} _{}^{}}{{\left( {770}\right) }{}_{}^{}}} ^{0}} = 773\pm 1$ $\,\text {(stat)}$ $\,\text {Me}\text {V}$ and $\varGamma _{{{{\uprho _{}^{}} _{}^{}}{{\left( {770}\right) }{}_{}^{}}} ^{0}} = 148\pm 3\,\text {(stat)} \,\text {Me}\text {V}$, and $M_{{{{\upomega _{}^{}}}{{\left( {783}\right) }{}_{}^{}}}} = 776\pm 2$ $\,\text {(stat)}$ $\,\text {Me}\text {V}$, consistent with the world average values [33]. The fitted value of the ${{\upomega _{}^{}}}{{\left( {783}\right) }{}_{}^{}}$ width, $\varGamma _{{{{\upomega _{}^{}}}{{\left( {783}\right) }{}_{}^{}}}} = 30\pm 5$ $\,\text {(stat)}$ $\,\text {Me}\text {V}$, is instead larger than the world average because of the detector resolution.

The $|B/A |$ and *C* / *A* fractions are also determined; they measure the ratios of the nonresonant and ${{\upomega _{}^{}}}{{\left( {783}\right) }{}_{}^{}}$ contributions to the resonant ${{{\uprho _{}^{}} _{}^{}}{{\left( {770}\right) }{}_{}^{}}} ^{0}$ production, respectively. Since the ZEUS Collaboration found that $|B/A |$ decreases as $|t |$ increases, the fit is repeated for $|t |<0.5$ $\text {Ge}\text {V} ^{2}$ resulting in $0.50 \pm 0.06$ $\,\text {(stat)}$ $\text {Ge}\text {V} ^{-1/2}$. For this kinematic region H1 measured $|B/A |=0.57 \pm 0.09$ $\,\text {(stat)}$ $\text {Ge}\text {V} ^{-1/2}$ and ZEUS $|B/A |=0.70 \pm 0.04$ $\,\text {(stat)}$ $\text {Ge}\text {V} ^{-1/2}$. If the fit is repeated without the ${{{\upomega _{}^{}}}{{\left( {783}\right) }{}_{}^{}}}-{{{\uprho _{}^{}} _{}^{}}{{\left( {770}\right) }{}_{}^{}}} ^{0}$ interference component, the result for $|B/A |$ changes by less than its statistical uncertainty. The measured ratio of the ${{\upomega _{}^{}}}{{\left( {783}\right) }{}_{}^{}}$ to ${{{\uprho _{}^{}} _{}^{}}{{\left( {770}\right) }{}_{}^{}}} ^{0}$ amplitudes is $C/A=0.40\pm 0.06$ $\,\text {(stat)}$, consistent with the prediction of starlight, $C/A=0.32$, and the measurements of the STAR [23] and the DESY-MIT [40] experiments, which report $C/A=0.36\pm 0.03$ $\,\text {(stat)}$ and $C/A=0.36\pm 0.04$ $\,\text {(stat)}$, respectively. The present fit gives a nonzero ${{\upomega _{}^{}}}{{\left( {783}\right) }{}_{}^{}}$ phase angle, $\phi _{{{\upomega _{}^{}}}{{\left( {783}\right) }{}_{}^{}}} =1.8\pm 0.3$ $\,\text {(stat)}$, also in agreement with the previous measurements [23, 40].

Additionally, the fit is performed in $|t |$ and *y* bins as shown in Fig. 4. To ensure fit stability, the $M_{{{{\uprho _{}^{}} _{}^{}}{{\left( {770}\right) }{}_{}^{}}} ^{0}}$, $M_{{{{\upomega _{}^{}}}{{\left( {783}\right) }{}_{}^{}}}}$, $\varGamma _{{{{\uprho _{}^{}} _{}^{}}{{\left( {770}\right) }{}_{}^{}}} ^{0}}$, $\varGamma _{{{{\upomega _{}^{}}}{{\left( {783}\right) }{}_{}^{}}}}$, $\phi _{{{\upomega _{}^{}}}{{\left( {783}\right) }{}_{}^{}}}$ and $|C/A |$ parameters are fixed to the values obtained for the full rapidity interval. The ${{{\upomega _{}^{}}}{{\left( {783}\right) }{}_{}^{}}} \rightarrow {{\uppi _{}^{}} _{}^{+}} {{\uppi _{}^{}} _{}^{-}} {{\uppi _{}^{}} _{}^{0}}$ contribution increases with $|t |$, as reported by the H1 Collaboration [18] and as seen in Fig. 4. The $|B/A |$ ratio is found to be independent of $W_{{\upgamma _{}^{}} \mathrm{p}}$ and decreases with $|t |$, in agreement with results reported by ZEUS [17].

## Systematic uncertainties

The following sources of systematic uncertainty are considered.

* Integrated luminosity determination*: The uncertainty in the integrated luminosity is 4% for both the pPb and Pbp samples [41].

* Track reconstruction*: The contribution of the tracking efficiency to the systematic uncertainty is studied with the method described in Ref. [26], where the ratio of yields of neutral charm mesons decaying to two-body and four-body final states is compared with data and simulation for pion momenta above 300$\,\text {Me}\text {V}$. The accuracy of the detector simulation to reproduce the single-pion tracking efficiency is 3.9%. For the present measurement, this yields a 7.8% uncertainty.

* Unfolding*: The uncertainty associated with the unfolding procedure is determined by modifying the number of iterations used for the Bayesian unfolding from the nominal value of 4 to 3 and 5. The resulting uncertainty is smaller than that found when changing the model for building the response matrix. The latter is estimated by comparing two different starlight samples: resonant ${{{\uprho _{}^{}} _{}^{}}{{\left( {770}\right) }{}_{}^{}}} ^{0}$ meson production, and combined resonant and nonresonant ${{\uppi _{}^{}} _{}^{+}} {{\uppi _{}^{}} _{}^{-}}$ production. The resulting effect on the integrated cross section is 3%.

* Uncertainty in the photon flux:* The uncertainty in the photon flux is 9% for the high-$W_{{\upgamma _{}^{}} \mathrm{p}}$ data point and 2% at low $W_{{\upgamma _{}^{}} \mathrm{p}}$, as discussed in Ref. [11]. The flux is computed in impact parameter space, convolved with the probability of no hadronic interactions. The radius of the lead nucleus is varied by the nuclear skin thickness ($\pm 0.5\,\text {fm}$). In addition, in the calculation of the photon flux, the ${{{\uprho _{}^{}} _{}^{}}{{\left( {770}\right) }{}_{}^{}}} ^{0}$ pole mass in Eq. (1) is replaced by the reconstructed ${{\uppi _{}^{}} _{}^{+}} {{\uppi _{}^{}} _{}^{-}}$ mass on an event-by-event basis. The effect of this variation is negligible.

* Calorimeter exclusivity*: The uncertainty related to the exclusivity requirements is evaluated by varying the calorimeter energy thresholds. Increasing (or decreasing) the energy scale of the HF calorimeter towers by 5% results in a 1.0% variation of the exclusive ${{\uppi _{}^{}} _{}^{+}} {{\uppi _{}^{}} _{}^{-}}$ yields. The CASTOR energy scale is varied by $17\%$ [42], resulting in a difference of 1% in the extracted ${{{\uprho _{}^{}} _{}^{}}{{\left( {770}\right) }{}_{}^{}}} ^{0}$ yield. The variations of the energy thresholds for HE and ZDC within their respective energy scale uncertainties have a negligible effect.

* Background estimation*: The uncertainty in the ${{{\uprho _{}^{}} _{}^{}}{{\left( {1700}\right) }{}_{}^{}}}$ subtraction is evaluated by varying the normalization of the ${{{\uprho _{}^{}} _{}^{}}{{\left( {1700}\right) }{}_{}^{}}}$ contribution by 20% with respect to that obtained from the fit shown in Fig. 2. As mentioned in Sect. 5, the proton dissociative background template is obtained by requiring a signal in at least one of the forward detectors: HF, CASTOR, or ZDC. To calculate the systematic uncertainty related to the estimation of this background, the analysis is repeated five times and each time alternative combinations of forward detectors are used to obtain the proton dissociative template. The following variations are studied: (i) HF alone; (ii) CASTOR alone; (iii) ZDC alone; (iv) HF or CASTOR; (v) HF or ZDC. For each of these combinations the proton dissociative contributions are obtained in each $|t |$ and rapidity bin. The maximum deviations from the nominal results are taken as conservative estimates of the systematic uncertainty. The resulting effect on the integrated exclusive ${{{\uprho _{}^{}} _{}^{}}{{\left( {770}\right) }{}_{}^{}}} ^{0}$ photoproduction cross section is smaller than 10%.

* Model dependence*: In order to assess the uncertainty due to the model used to fit the invariant mass distribution, the Ross–Stodolsky model [43] is used instead of the Söding model. The resulting cross section changes by up to 8%, depending on the rapidity and $|t |$ interval studied. Another contribution to the model dependence uncertainty comes from the reweighting procedure of the starlight MC described in Sect. 6. This uncertainty is evaluated by varying the reweighting parameter *b* within its uncertainty; it is found to increase as a function of $|t |$, and reaches 32% for the highest $|t |$ bin. The second contribution turns out to be dominant for all the rapidity and $|t |$ intervals studied. The uncertainty in the extrapolation to the region $|t | < 0.025\,\text {Ge}\text {V} ^{2}$ is model dependent. We estimated this uncertainty by studying different fitting functions to the differential cross section measurements. In particular, we studied a dipole form [28], a pure exponential $\mathrm {e}^{-bt}$, and a modified exponential $\mathrm {e}^{-bt+ct^2}$. The difference between the two most extreme extrapolated values is used as an estimate of the model dependence uncertainty.

The values of the systematic uncertainties for all $y_{{{\uppi _{}^{}} _{}^{+}} {{\uppi _{}^{}} _{}^{-}}}$ and $|t |$ intervals are summarized in Table 2. The systematic uncertainties are added in quadrature for the integrated photoproduction cross section. For the differential cross section results, the systematic uncertainties in Table 2 are treated as correlated between bins.

**Table 2:** Summary of the systematic uncertainties in the ${{{\uprho _{}^{}} _{}^{}}{{\left( {770}\right) }{}_{}^{}}} ^{0}$ photoproduction cross section. The numbers are given in percent. The total uncertainty is calculated by adding the individual uncertainties in quadrature

$y_{{{\uppi _{}^{}} _{}^{+}} {{\uppi _{}^{}} _{}^{-}}}$ interval	$(-2.0, 2.0)$	$(-2.0, -1.2)$	$(-1.2, 0.0)$	(0.0, 1.2)	(1.2, 2.0)
Integrated luminosity	4.0	4.0	4.0	4.0	4.0
Track reconstruction	7.8	7.8	7.8	7.8	7.8
Unfolding	3.0	3.0	3.0	3.0	3.0
Photon flux calculation	5.0	2.0	4.0	6.0	9.0
Calorimeter exclusivity	1.4	1.4	1.4	1.4	1.4
Proton dissociation					
$$|t | [\text {Ge}\text {V} ^{2}]$$					
0.025−1.000	2.3	1.6	2.3	3.6	3.9
0.025−0.075	2.3	1.6	2.3	3.6	3.9
0.075−0.120	1.9	1.5	1.8	2.9	3.2
0.12−0.17	2.3	1.7	2.1	3.3	3.7
0.17−0.24	3.0	2.2	2.7	4.0	4.9
0.24−0.30	3.9	2.5	3.4	5.5	6.8
0.3−0.4	5.2	3.7	4.6	7.1	9.2
0.40−0.55	7.1	5.8	6.5	9.8	13.0
0.55−0.75	10.0	9.7	9.0	14.0	19.0
0.75−1.00	14.0	19.0	11.0	22.0	28.0
${{{\uprho _{}^{}} _{}^{}}{{\left( {1700}\right) }{}_{}^{}}}$ background					
$$|t | [\text {Ge}\text {V} ^{2}]$$					
0.025−1.000	4.3	13.0	1.9	7.1	2.0
0.025−0.075	4.3	13.0	1.9	7.1	2.0
0.075−0.120	4.8	1.3	2.0	2.0	7.4
0.12−0.17	5.6	2.9	2.7	4.5	5.6
0.17−0.24	5.8	3.6	5.9	3.2	4.9
0.24−0.30	3.8	4.4	6.6	5.8	16.0
0.3−0.4	6.5	14.0	7.3	11.0	17.0
0.40−0.55	9.1	19.0	21.0	9.7	14.0
0.55−0.75	35.0	37.0	13.0	20.0	55.0
0.75−1.00	46.0	56.0	19.0	39.0	32.0
Model dependence					
$$|t | [\text {Ge}\text {V} ^{2}]$$					
0.025−1.000	5.1	5.1	5.1	5.1	5.1
0.025−0.075	5.1	5.1	5.1	5.1	5.1
0.075−0.120	5.5	5.5	5.5	5.5	5.5
0.12−0.17	6.6	6.6	6.6	6.6	6.5
0.17−0.24	8.6	8.6	8.6	8.6	8.6
0.24−0.30	12.0	12.0	12.0	12.0	11.0
0.3−0.4	15.0	16.0	16.0	15.0	15.0
0.40−0.55	20.0	20.0	20.0	20.0	20.0
0.55−0.75	26.0	26.0	26.0	26.0	25.0
0.75−1.00	32.0	32.0	32.0	32.0	32.0

## Results

The differential cross section for exclusive photoproduction of ${{{\uprho _{}^{}} _{}^{}}{{\left( {770}\right) }{}_{}^{}}} ^{0}$ mesons is given by

$$\begin{aligned} \frac{\mathrm {d}\sigma }{\mathrm {d}y} = \frac{N^{\text {exc}}_{{{{\uprho _{}^{}} _{}^{}}{{\left( {770}\right) }{}_{}^{}}} ^{0}}}{{\mathcal {B}}({{{\uprho _{}^{}} _{}^{}}{{\left( {770}\right) }{}_{}^{}}} ^{0}\rightarrow {\uppi _{}^{}} ^{+}{\uppi _{}^{}} ^{-}) L \varDelta y}, \end{aligned}$$

where $N^{\text {exc}}_{{{{\uprho _{}^{}} _{}^{}}{{\left( {770}\right) }{}_{}^{}}} ^{0}}$ is the corrected number of exclusive ${{{\uprho _{}^{}} _{}^{}}{{\left( {770}\right) }{}_{}^{}}} ^{0}$ events obtained from the fits described in Sect. 6 by integrating the resonant component in the interval $0.28< M_{{{{\uprho _{}^{}} _{}^{}}{{\left( {770}\right) }{}_{}^{}}} ^{0}}<1.50\,\text {Ge}\text {V}$ ($2M_{{{\uppi _{}^{}} _{}^{\pm }}}< M_{{{{\uprho _{}^{}} _{}^{}}{{\left( {770}\right) }{}_{}^{}}} ^{0}} < M_{{{{\uprho _{}^{}} _{}^{}}{{\left( {770}\right) }{}_{}^{}}} ^{0}} + 5\varGamma _{{{{\uprho _{}^{}} _{}^{}}{{\left( {770}\right) }{}_{}^{}}} ^{0}}$); ${\mathcal {B}}$ is the branching fraction, which equals about 0.99 for the ${{{\uprho _{}^{}} _{}^{}}{{\left( {770}\right) }{}_{}^{}}} ^{0}\rightarrow {\uppi _{}^{}} ^{+}{\uppi _{}^{}} ^{-}$ decay [33], $\varDelta y$ is the rapidity interval, and *L* is the integrated luminosity of the data sample. The cross section $\mathrm {d}\sigma /\mathrm {d}y(\mathrm{p}\mathrm {Pb}\rightarrow \mathrm{p}\mathrm {Pb}{{{\uprho _{}^{}} _{}^{}}{{\left( {770}\right) }{}_{}^{}}} ^{0})$ is related to the photon–proton cross section, $\sigma ({\upgamma _{}^{}} \mathrm{p}\rightarrow {{{\uprho _{}^{}} _{}^{}}{{\left( {770}\right) }{}_{}^{}}} ^{0}\mathrm{p}) \equiv \sigma (W_{{\upgamma _{}^{}} \mathrm{p}})$, through the photon flux, $\mathrm {d}n/\mathrm {d}k$:

$$\begin{aligned} \frac{\mathrm {d}\sigma }{\mathrm {d}y}(\mathrm{p}\mathrm {Pb}\rightarrow \mathrm{p}\mathrm {Pb}{{{\uprho _{}^{}} _{}^{}}{{\left( {770}\right) }{}_{}^{}}} ^{0}) = k \frac{\mathrm {d}n}{\mathrm {d}k} \sigma ({\upgamma _{}^{}} \mathrm{p}\rightarrow {{{\uprho _{}^{}} _{}^{}}{{\left( {770}\right) }{}_{}^{}}} ^{0}\mathrm{p}). \end{aligned}$$

Here, *k* is the photon energy, which is determined from the ${{{\uprho _{}^{}} _{}^{}}{{\left( {770}\right) }{}_{}^{}}} ^{0}$ mass and rapidity, according to the formula

1 $$\begin{aligned} k = (1/2) M_{{{{\uprho _{}^{}} _{}^{}}{{\left( {770}\right) }{}_{}^{}}} ^{0}} \exp {(-y_{{{{\uprho _{}^{}} _{}^{}}{{\left( {770}\right) }{}_{}^{}}} ^{0}})}. \end{aligned}$$

The average photon flux and the average centre-of-mass energy ($\langle W_{{\upgamma _{}^{}} \mathrm{p}} \rangle$) values in each rapidity interval are calculated using starlight.

**Table 3:** Differential cross section for exclusive ${{{\uprho _{}^{}} _{}^{}}{{\left( {770}\right) }{}_{}^{}}} ^{0}$ photoproduction, $\sigma ({\upgamma _{}^{}} \mathrm{p}\rightarrow {{{\uprho _{}^{}} _{}^{}}{{\left( {770}\right) }{}_{}^{}}} ^{0}\mathrm{p})$, with statistical and systematic uncertainties, for $|t |<0.5$ $\text {Ge}\text {V} ^{2}$. The differential cross section $\mathrm {d}\sigma /\mathrm {d}|t |$ is also shown, along with the rapidity range, the average value of $W_{{\upgamma _{}^{}} \mathrm{p}}$, $\langle W_{{\upgamma _{}^{}} \mathrm{p}}\rangle$, and $k \frac{\mathrm {d}n}{\mathrm {d}k}$

*y* range	(-2.0, 2.0)	(-2.0, -1.2)	(-1.2, 0.0)	(0.0, 1.2)	(1.2, 2.0)
$W_{{\upgamma _{}^{}} \mathrm{p}}$ range [$\text {Ge}\text {V}$ ]	(29, 213)	(29, 43)	(43, 78)	(78, 143)	(143, 213)
$\langle W_{{\upgamma _{}^{}} \mathrm{p}}\rangle$ [$\text {Ge}\text {V}$ ]	92.6	35.6	59.2	108.0	176.0
$k \frac{\mathrm {d}n}{\mathrm {d}k}$	136.0	186.0	155.0	117.0	86.2
$\mathrm {d}\sigma /\mathrm {d}y$ [$\mu$b]	11.0	9.1	9.9	12.4	12.9
Stat. unc. [$\mu$b]	1.4	1.5	1.6	2.4	2.6
Syst. unc. [$\mu$b]	1.0	0.8	0.9	1.1	1.3
$$|t | $$[$$\text {Ge}\text {V}$$ $$^{2}$$]	$$\mathrm {d}\sigma /\mathrm {d}|t |$$ [$$\mu $$b/$$\text {Ge}\text {V}$$ $$^{2}$$]	$$\mathrm {d}\sigma /\mathrm {d}|t |$$ [$$\mu $$b/$$\text {Ge}\text {V}$$ $$^{2}$$]	$$\mathrm {d}\sigma /\mathrm {d}|t |$$ [$$\mu $$b/$$\text {Ge}\text {V}$$ $$^{2}$$]	$$\mathrm {d}\sigma /\mathrm {d}|t |$$ [$$\mu $$b/$$\text {Ge}\text {V}$$ $$^{2}$$]	$$\mathrm {d}\sigma /\mathrm {d}|t |$$ [$$\mu $$b/$$\text {Ge}\text {V}$$ $$^{2}$$]
0.025−0.075	$56.0\pm 2.2\pm 6.4$	$47.0\pm 4.5\pm 4.9$	$50.0\pm 4.1\pm 5.5$	$57.7\pm 6.1\pm 6.9$	$74.5\pm 7.9\pm 10.2$
0.075−0.125	$33.6\pm 1.0\pm 3.9$	$26.0\pm 2.3\pm 2.8$	$30.2\pm 1.9\pm 3.4$	$39.1\pm 3.2\pm 4.7$	$39.3\pm 3.4\pm 5.5$
0.125−0.175	$24.4\pm 0.8\pm 3.0$	$22.1\pm 2.1\pm 2.6$	$18.8\pm 1.2\pm 2.3$	$24.3\pm 2.2\pm 3.1$	$26.6\pm 2.3\pm 3.9$
0.175−0.240	$15.5\pm 0.7\pm 2.1$	$10.9\pm 1.3\pm 1.4$	$14.6\pm 1.2\pm 2.0$	$16.5\pm 1.9\pm 2.4$	$14.1\pm 1.7\pm 2.2$
0.24−0.30	$10.2\pm 0.6\pm 1.6$	$6.7\pm 0.8\pm 1.0$	$9.7\pm 0.9\pm 1.5$	$11.8 \pm 1.9\pm 1.9$	$8.1\pm 1.1\pm 1.4$
0.3−0.4	$5.2\pm 0.4\pm 1.4$	$5.0\pm 0.9\pm 1.4$	$4.0\pm 0.5\pm 1.4$	$6.6\pm 1.5\pm 1.4$	$3.3\pm 0.6\pm 1.4$
0.40−0.55	$3.5\pm 0.4\pm 1.4$	$2.2\pm 0.6\pm 1.4$	$3.4\pm 0.6\pm 1.4$	$3.0\pm 1.0\pm 1.4$	$1.9\pm 0.5\pm 1.4$
0.55−0.75	$1.4\pm 0.3\pm 1.4$	$0.94\pm 0.44\pm 1.4$	$1.5\pm 0.3\pm 1.4$	$1.2\pm 0.6\pm 1.4$	$1.0\pm 0.3\pm 1.4$
0.75−1.00	$0.52\pm 0.14\pm 1.4$	$0.37\pm 0.28\pm 1.4$	$0.50\pm 0.12\pm 1.4$	$0.60\pm 0.47\pm 1.4$	$0.38\pm 0.22\pm 1.4$

**Fig. 5:**
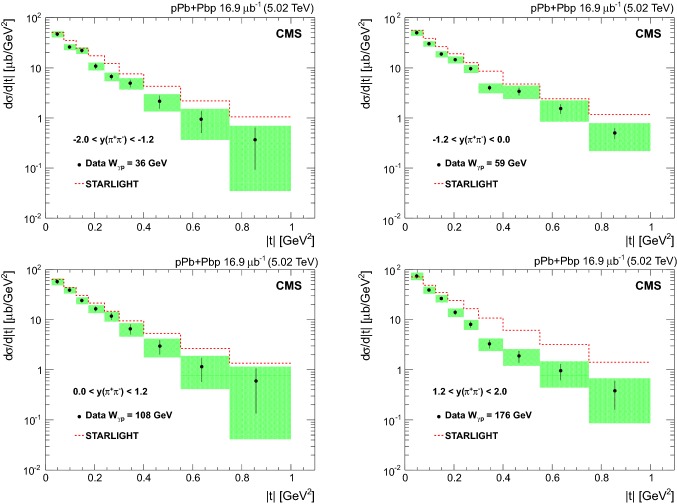
Differential cross section $\mathrm {d}\sigma /\mathrm {d}|t |$ (full circles) in four different rapidity bins. The error bars show the statistical uncertainty, whereas the shaded areas represent the statistical and systematic uncertainties added in quadrature. The dashed lines show the unweighted starlight predictions

The unfolded invariant mass distribution is studied in different $|t |$ bins, and the extraction of the ${{{\uprho _{}^{}} _{}^{}}{{\left( {770}\right) }{}_{}^{}}} ^{0}$ photoproduction cross section is performed in each bin. In order to compare with the HERA results, the $p_{\mathrm {T}}$-related measurements are presented in terms of $|t |$, which is approximated as $|t | \approx (p_{\mathrm {T}} ^{{{\uppi _{}^{}} _{}^{+}} {{\uppi _{}^{}} _{}^{-}}})^{2}$. Figure 5 shows the differential cross sections as a function of $|t |$, together with the unweighted starlight prediction, whose slope parameter is independent of $W_{{\upgamma _{}^{}} \mathrm{p}}$. The starlight prediction is systematically higher than the data in the high-$|t |$ region. This trend becomes more significant as $W_{{\upgamma _{}^{}} \mathrm{p}}$ increases.

**Fig. 6:**
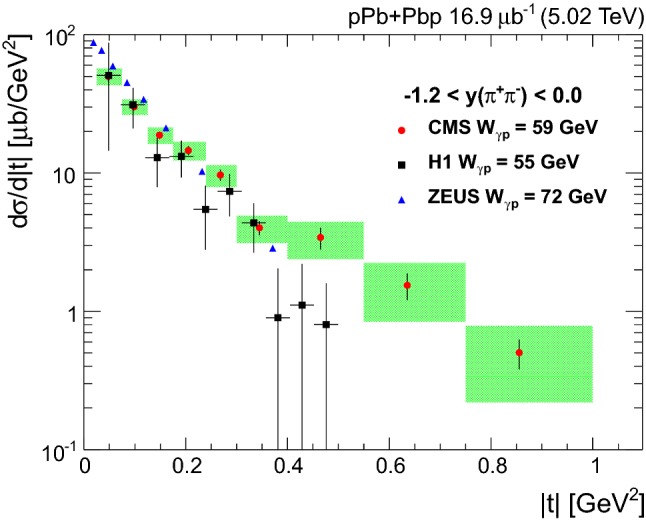
Differential cross section $\mathrm {d}\sigma /\mathrm {d}|t |$ (full circles) for exclusive ${{{\uprho _{}^{}} _{}^{}}{{\left( {770}\right) }{}_{}^{}}} ^{0}$ photoproduction in the rapidity interval $-1.2< y_{{{\uppi _{}^{}} _{}^{+}} {{\uppi _{}^{}} _{}^{-}}} < 0$. The square symbols indicate the H1 results, and the triangles the ZEUS results. The error bars show the statistical uncertainty, while the shaded areas represent the statistical and systematic uncertainties added in quadrature. For the H1 data [18], the error bars represent the statistical and systematic uncertainties added in quadrature, and for the ZEUS data [17] the reported uncertainties are negligible

Figure 6 shows the differential cross section $\mathrm {d}\sigma /\mathrm {d}|t |$ in the rapidity interval $-1.2< y({{\uppi _{}^{}} _{}^{+}} {{\uppi _{}^{}} _{}^{-}}) < 0$ compared with the H1 and ZEUS results [17, 18] in a similar $W_{{\upgamma _{}^{}} \mathrm{p}}$ range.

**Fig. 7:**
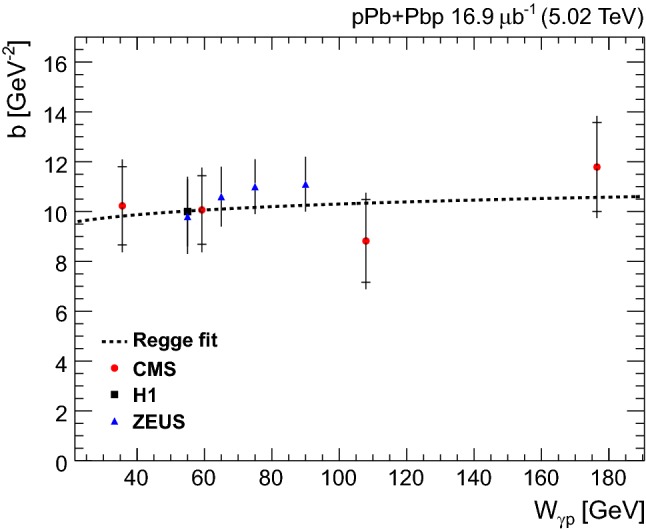
The slope parameter *b* extracted from the exponential fits of the differential cross sections $\mathrm {d}\sigma /\mathrm {d}|t |$ shown as a function of $W_{{\upgamma _{}^{}} \mathrm{p}}$. The inner error bars show the statistical uncertainty, while the outer error bars indicate the statistical and systematic uncertainties added in quadrature. The dashed line shows the result of the Regge fit discussed in the text

The differential cross section as a function of $|t |$ is fitted with the form $A\mathrm {e}^{-bt+ct{^2}}$ in the region $0.025<|t |<0.5$ $\text {Ge}\text {V} ^{2}$. For the integrated rapidity bin the fit gives $b=9.2\pm 0.7$ $\,\text {(stat)}$ $\text {Ge}\text {V} ^{-2}$ and $c=4.6\pm 1.6$ $\,\text {(stat)}$ $\text {Ge}\text {V} ^{-4}$. The resulting values of the slope *b* are shown in Fig. 7 as a function of $W_{{\upgamma _{}^{}} \mathrm{p}}$, together with those measured by H1 and ZEUS [17, 18]. The values of the parameter *c* are found to be constant within the fit uncertainties. The Regge formula [44] $b = b_{0} + 2\alpha '\ln (W_{{\upgamma _{}^{}} \mathrm{p}}/W_{0})^{2}$, which parametrizes the dependence of *b* on the collision energy, is fitted to the data using $W_{0} = 92.6$ $\,\text {Ge}\text {V}$, the average centre-of-mass energy of the present data. The fit to the CMS data alone gives a pomeron slope of $\alpha ' = 0.28 \pm 0.11\,\text {(stat)} \pm 0.12$ $\,\text {(syst)}$ $\text {Ge}\text {V} ^{-2}$, consistent with the ZEUS [17] value and the Regge expectation of $0.25~\text {Ge}\text {V} ^{-2}$.

**Fig. 8:**
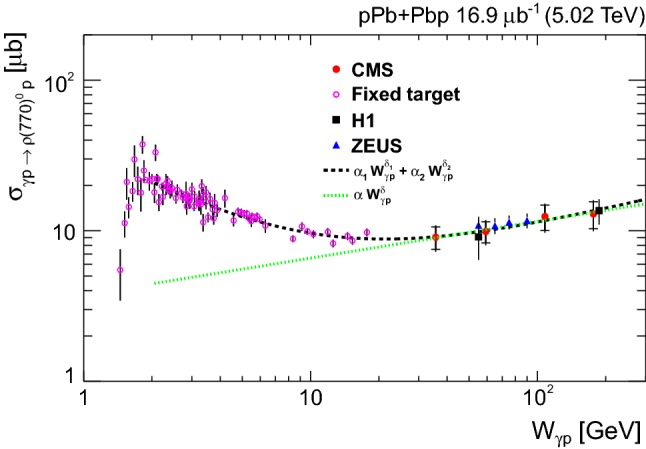
Exclusive ${{{\uprho _{}^{}} _{}^{}}{{\left( {770}\right) }{}_{}^{}}} ^{0}$ photoproduction cross section as a function of $W_{{\upgamma _{}^{}} \mathrm{p}}$. The inner bars show the statistical uncertainty, while the outer bars represent the statistical and systematic uncertainties added in quadrature. Fixed-target [45–48] and HERA [17, 18] data are also shown. The dashed lines indicate the results of the fits described in the text

The resulting photon–proton cross section, obtained for $W_{{\upgamma _{}^{}} \mathrm{p}}$ between 29 and 213$\,\text {Ge}\text {V}$ ($\langle W_{{\upgamma _{}^{}} \mathrm{p}} \rangle = 92.6$ $\,\text {Ge}\text {V}$) is extrapolated to the range $0<|t |<0.5~\text {Ge}\text {V} ^{2}$ using the exponential fits just discussed and the starlight predictions in order to allow direct comparison with previous experiments. The resulting value is $\sigma = 11.0 \pm 1.4$ $\,\text {(stat)}$ $\pm 1.0$ $\,\text {(syst)}$ $\mu$b. The photon–proton cross section values, $\sigma ({\upgamma _{}^{}} \mathrm{p}\rightarrow {{{\uprho _{}^{}} _{}^{}}{{\left( {770}\right) }{}_{}^{}}} ^{0}\mathrm{p})$, for all rapidity bins are presented in Table 3 and Fig. 8. Figure 8 also shows a compilation of fixed-target [45–48] and HERA results [17, 18]. The results of two fits are shown in Fig. 8. The dashed line indicates the result of a fit to all the plotted data with the formula $\sigma = \alpha _{1} W^{\delta _{1}}_{{\upgamma _{}^{}} \mathrm{p}} + \alpha _{2} W^{\delta _{2}}_{{\upgamma _{}^{}} \mathrm{p}}$ (see e.g. [19, 20]). The fit describes the data well and yields the values $\delta _{1} = -0.81 \pm 0.04$ $\,\text {(stat)}$ $\pm 0.09$ $\,\text {(syst)}$, $\delta _{2} = 0.36 \pm 0.07$ $\,\text {(stat)}$ $\pm 0.05$ $\,\text {(syst)}$. The CMS and HERA data are also fitted with the function $\sigma = \alpha W^{\delta }_{{\upgamma _{}^{}} \mathrm{p}}$ as shown in Fig. 8. The fit yields $\delta = 0.24 \pm 0.13$ $\,\text {(stat)}$ $\pm 0.04$ $\,\text {(syst)}$. Only statistical and uncorrelated systematic uncertainties are considered in these fits.

## Summary

The CMS Collaboration has made the first measurement of exclusive ${{{\uprho _{}^{}} _{}^{}}{{\left( {770}\right) }{}_{}^{}}} ^{0}$ photoproduction off protons in ultraperipheral pPb collisions at $\sqrt{\smash [b]{s_{_{\mathrm {NN}}}}} = 5.02\,\text {Te}\text {V}$. The cross section for this process is measured in the photon–proton centre-of-mass energy interval $29< W_{{\upgamma _{}^{}} \mathrm{p}} < 213\,\text {Ge}\text {V}$. The results are consistent with those of the H1 and ZEUS Collaborations at HERA, indicating that ion–proton collisions can be used in the same way as electron–proton ones, with ions acting as a source of quasi-real photons. The combination of the present data and the earlier, lower energy results agrees with theory-inspired fits. The differential cross section $\mathrm {d}\sigma /\mathrm {d}|t |$ for ${{{\uprho _{}^{}} _{}^{}}{{\left( {770}\right) }{}_{}^{}}} ^{0}$ photoproduction is measured as a function of $W_{{\upgamma _{}^{}} \mathrm{p}}$. The starlight prediction is systematically higher than the data in the high-$|t |$ region. This trend becomes more significant as $W_{{\upgamma _{}^{}} \mathrm{p}}$ increases.

## Data Availability

This manuscript has no associated data or the data will not be deposited. [Authors’ comment: Release and preservation of data used by the CMS Collaboration as the basis for publications is guided by the CMS policy as written in its document “CMS data preservation, re-use and open access policy” (https://cmsdocdb.cern.ch/cgi-bin/PublicDocDB/RetrieveFile?docid=6032&filename=CMSDataPolicyV1.2.pdf&version=2).]

## Footnotes

1 The data on the photoproduction of excited ${{{\uprho _{}^{}} _{}^{}}{{\left( {1700}\right) }{}_{}^{}}}$ states in the four-pion decay channel are currently limited. A resonance structure with a broad invariant mass distribution around 1600$\,\text {Me}\text {V}$ is reported in the literature. According to the Particle Data Group this resonance has two components: the ${{{\uprho _{}^{}} _{}^{}}{{\left( {1450}\right) }{}_{}^{}}}$ and the ${{{\uprho _{}^{}} _{}^{}}{{\left( {1700}\right) }{}_{}^{}}}$ [33]. The nature of these states is still under investigation. Recently, the STAR Collaboration reported a measurement of exclusive photoproduction of four charged pions [34]. Their data are consistent with the ${{{\uprho _{}^{}} _{}^{}}{{\left( {1700}\right) }{}_{}^{}}}$ assuming that the peak is dominated by spin states with $J^{PC} = 1^{--}$. In order to reproduce these data, starlight assumes a single resonance with a mass of 1540$\,\text {Me}\text {V}$ and a width of 570$\,\text {Me}\text {V}$ [28]. In the present paper, this state is referred to as ${{{\uprho _{}^{}} _{}^{}}{{\left( {1700}\right) }{}_{}^{}}}$.
